# Innovations in Printed Transparent Conducting Electrodes for Advanced Next‐Generation Electronics: A Comprehensive Review

**DOI:** 10.1002/smll.75727

**Published:** 2026-09-23

**Authors:** Vinayak Vitthal Satale, Sourav Acharya, Keum‐Jin Ko, Sagnik Chowdhury, Danya Srinivasan, Abrar Karim, Jae‐Wook Kang

**Affiliations:** ^1^ Department of Flexible and Printable Electronics LANL‐CBNU Engineering Institute, Korea Jeonbuk National University Jeonju Republic of Korea; ^2^ The Organic Photonics and Electronics Group Department of Physics Umeå University Umeå Sweden

**Keywords:** advanced printing processing, flexibility, photovoltaics, sensors, supercapacitors, transparent conducting electrodes

## Abstract

The emergence of wearable electronics has revolutionized photophysical and electrochemical, enabling lightweight, flexible energy harvesting that transcends the limitations of conventional rigid electronic technologies. With power conversion efficiencies now approaching those of established silicon devices, emerging electronics (photovoltaics, photodetectors, light‐emitting diodes, and energy storage devices) stand at the forefront of next‐generation wearable technology. Advancing high‐performance, flexible, next‐generation devices depends on developing transparent electrodes that successfully integrate excellent electrical conductivity, outstanding optical transparency, strong mechanical flexibility, and ultrathin form factors. This review explores how the figure of merit serves as a multidimensional measure of the optical, electrical, and mechanical properties of transparent electrodes. We provide an in‐depth analysis of cutting‐edge transparent conductive electrode platforms spanning conducting polymers, metallic nanowire networks and mesh architectures, carbon‐based nanomaterials, transparent conducting oxides, and emerging novel materials, each offering unique advantages for flexible electronic device applications. Looking ahead, we critically evaluate the technological challenges and transformative opportunities shaping the pathway from laboratory innovations to commercially viable, flexible, and wearable electronics, ultimately enabling a new era of adaptable, sustainable energy solutions.

## Graphical abstract

This review highlights advances in transparent conducting electrodes for flexible wearable electronics, analyzing their optical, electrical, and mechanical properties and their prospects for sustainable, next‐generation energy devices.

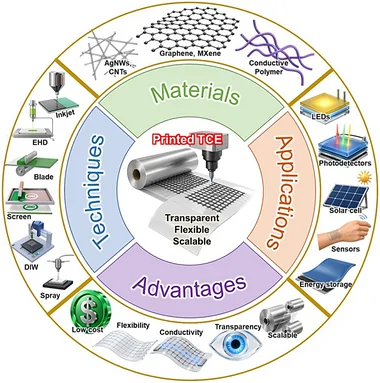

## 1 Introduction

Transparent conductive electrodes (TCEs) play a vital role in emerging electronics, including photovoltaics (PV), photodetectors (PD), light‐emitting diodes (LEDs), and energy storage devices [1, 2, 3, 4, 5, 6, 7, 8]. Indium tin oxide (ITO) serves as a widely utilized transparent electrode due to its excellent transmittance, electrical conductivity, and stability [9, 10, 11, 12, 13, 14, 15, 16, 17, 18, 19]. Due to the use of the scarce element indium, the large‐scale industrialization of the device may face bottlenecks. To find alternative transparent electrode production to reduce the consumption of depletable indium sources, some types of transparent electrodes are under development, for instance, graphene [20, 21], carbon nanotubes [22, 23, 24], PEDOT:PSS [19, 20, 25, 26, 27, 28, 29], metal nanowires [18, 30, 31, 32, 33, 34, 35, 36, 37, 38], thin metal films [39, 40, 41, 42, 43], metal meshes [6, 14, 44, 45, 46, 47, 48], metallic nanofibers [24, 49, 50, 51], and combinations of some of them [23, 52, 53]. Among the above‐mentioned transparent electrodes, the metal mesh has been widely explored due to its excellent conductivity and good mechanical properties [6, 45, 46]. A metal grid pattern is made up of interconnected metal lines arranged in a mesh‐like structure. This configuration provides efficient electrical conductivity while preserving transparency in areas without metal. This provides greater stability than certain other flexible electrodes. There are several techniques that have been used to pattern the metal grid electrode, including E‐beam lithography [54], imprinting [55, 56], laser writing [57], electrodeposition [58, 59], three‐dimensional (3D) printing [60, 61, 62, 63, 64, 65], and inkjet printing [66, 67, 68, 69]. In perovskite solar cells (PSCs), metal meshes are incorporated into the front and back electrodes to fulfill different purposes. The bottom gold (Au) mesh contact serves as a substitute for the traditional ITO electrode, which has drawbacks including environmental concerns, reduced efficiency at scale, and limited indium availability. On the other hand, integrating a silver (Ag) mesh as the top contact can improve device performance by supporting bifacial or multi‐junction setups. The selection of materials and fabrication methods for these metal meshes reflects their intended role. Gold is used for the rear electrode because it is well‐suited for interacting with the perovskite layers. Fine‐mesh patterns are fabricated via UV photolithography to optimize both electrical conductivity and optical transparency, a critical consideration for *p‐i‐n* devices that require the rear electrode to transmit light. For the top electrode, silver is chosen over gold because it does not directly contact the perovskite layer, thereby eliminating the risk of ion migration. Silver meshes are also favored for their higher conductivity and lower cost [6].

In this review, we present the latest advancements and technologies in emerging electronic devices, including PSCs, organic solar cells (OSCs), PDs, organic (O)‐LEDs, perovskite quantum dot (PeQ)‐LEDs, and energy storage devices such as supercapacitors, batteries, and electrochromic systems. Section one provides an overview of the materials and methods used to achieve transparent electrodes in these devices. Additionally, several reviews offer a comprehensive perspective on the fundamental properties and fabrication techniques of TCEs [6, 8, 42, 70, 71, 72, 73]. This review thoroughly analyzes the advantages and disadvantages of various TCEs from the perspective of the unique requirements of transparent optoelectronic devices, including compatibility with fabrication processes and electrode integration. We present a comprehensive overview of widely used TCEs in optoelectronics, including metals, metal oxides, conducting polymers, carbon materials, and composites, along with their respective preparation methods as illustrated in Figure 1. The following sections highlight recent advancements in high‐performance optoelectronic devices. Despite significant progress, research on transparent optoelectronic devices continues to evolve. We conclude by identifying key challenges in current technologies and proposing strategies to advance the fabrication of highly efficient, transparent, and flexible emerging electronic devices. It is anticipated that this review will stimulate increased engagement among both academic and industrial researchers, thereby facilitating advancements in see‐through display technologies, energy storage, and building‐integrated photovoltaics.

**FIGURE 1:**
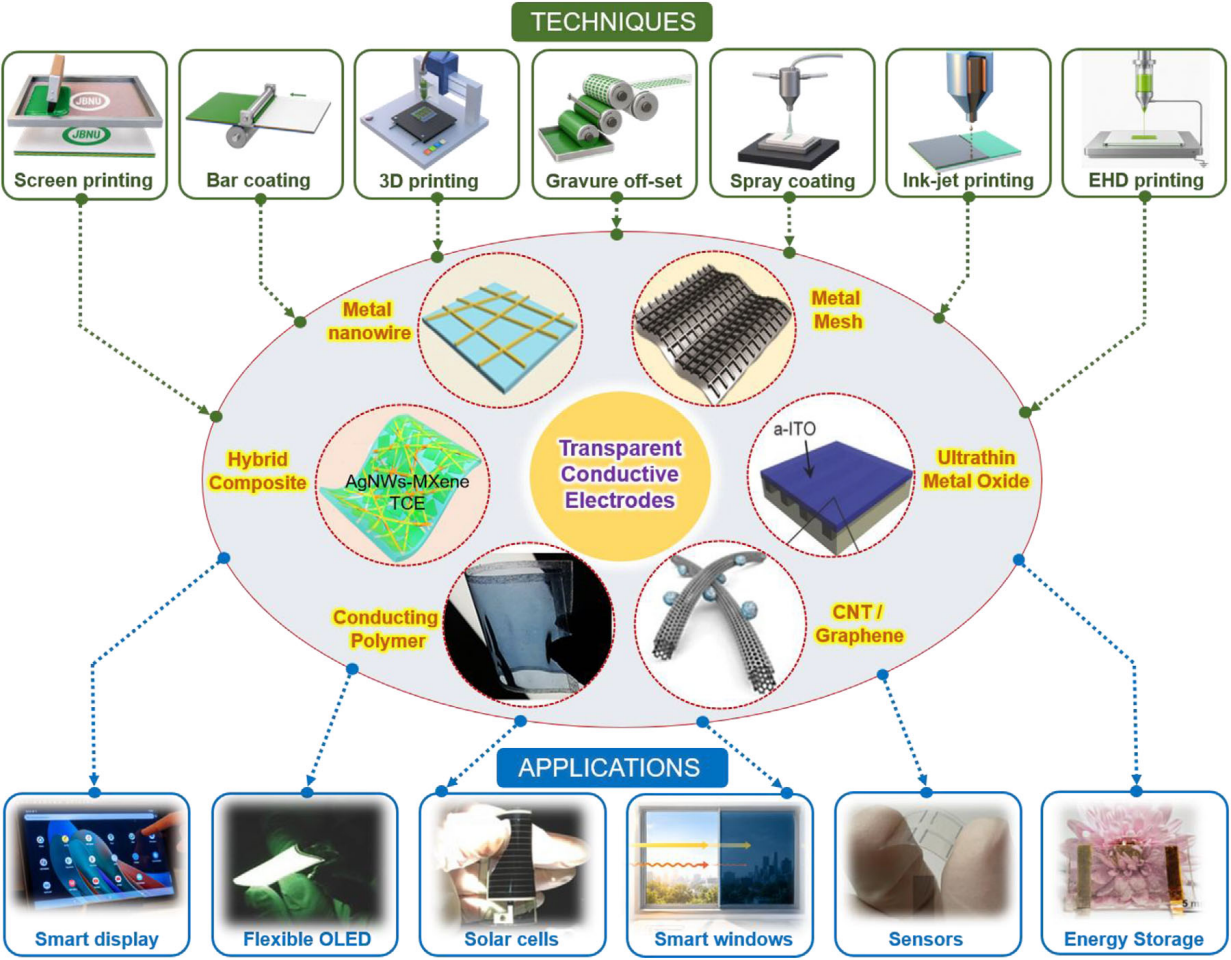
Schematic of the different types of TCEs, fabrication techniques, and their emerging electronic device applications. Reproduced with permission [41]. Copyright 2028, American Chemical Society. Reproduced with permission [6]. Copyright 2019, Royal Society of Chemistry. Reproduced with permission [74]. Copyright 2014, John Wiley and Sons. Reproduced with permission [75]. Copyright 2020, Elsevier. Reproduced with permission [76]. Copyright 2022, John Wiley and Sons. Reproduced with permission [77]. Copyright 2021, Elsevier. Reproduced with permission [78]. Copyright 2024, John Wiley and Sons. Reproduced with permission [79]. Copyright 2022. Royal Society of Chemistry.

## 2 Overview of Transparent Conducting Electrodes (TCEs)

This section provides an overview of the properties and fabrication methods of key flexible TCEs used in emerging electronic devices. Section 2.1 covers the assessment of TCEs, including essential formulas and expressions. Section 2.2 outlines the unique characteristics of TCEs. Section 2.3 discusses the distinguishing features and benefits of TCEs. Finally, Section 2.4 categorizes TCEs according to the materials used in their fabrication.

### 2.1 Assessments of the TCEs

Optimizing device performance requires that a TCE achieves a suitable balance between its optical transparency and sheet resistance. TCEs offer a strong balance between performance and cost, making them a prominent choice compared to other ITO alternatives, as shown schematically in Figure 2a. The effectiveness of a TCE is often evaluated using a figure of merit (*FoM*), a parameter that summarizes its optical and electrical characteristics. One of the earliest and most widely used FoM expressions for TCEs, introduced by Haacke, incorporates both the transmittance at 550 nm (T_550nm_) and the sheet resistance (*R_sheet_*) of the material, as shown in the following equation [80]:

(1) $$\Phi_{\mathrm{TC}}=\frac{T_{550\;nm}^{10}}{R_{sheet}\left(\Omega^{-1}\right)}\;$$

**FIGURE 2:**
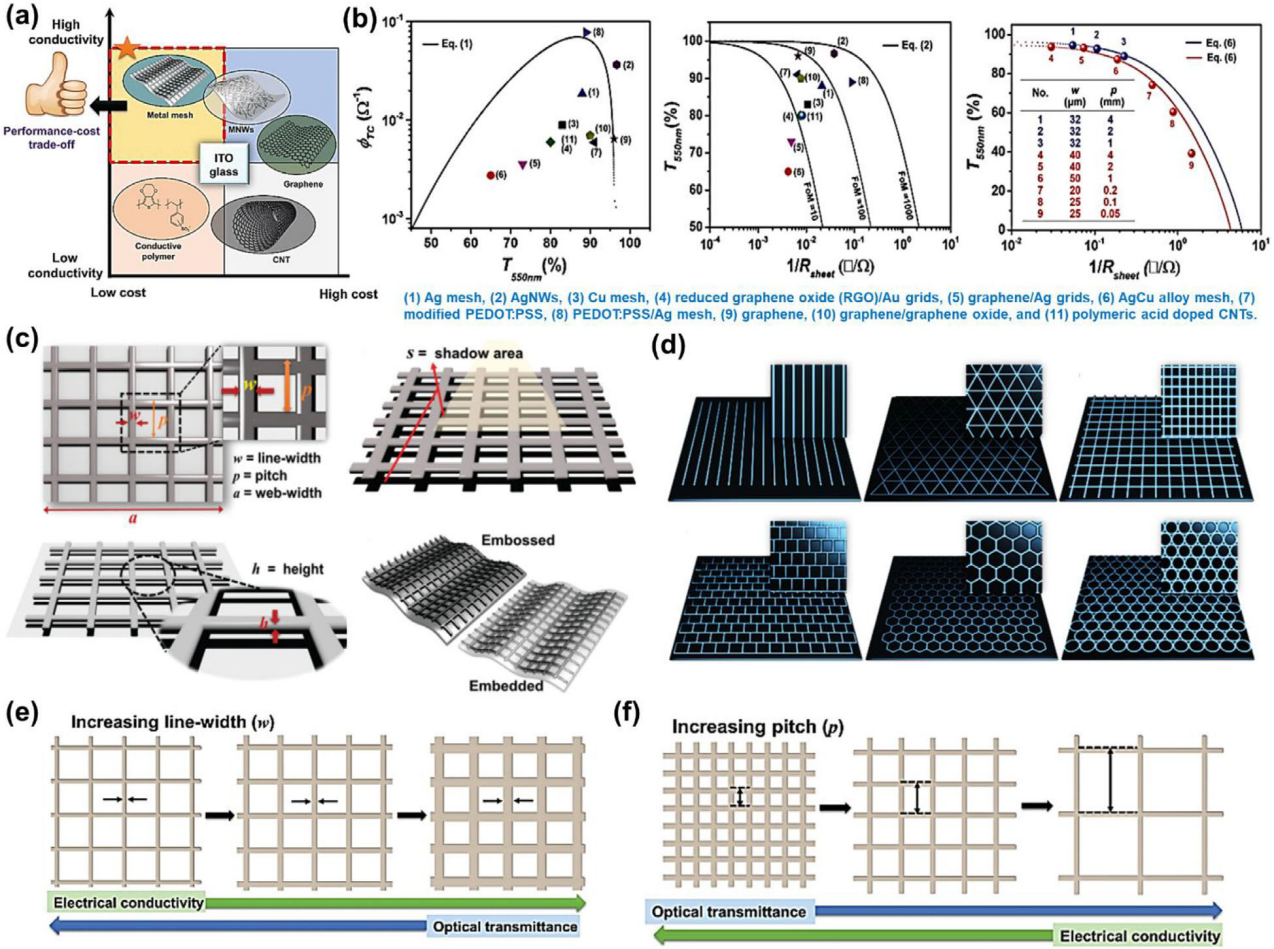
(a) Illustration comparing the performance and fabrication costs between traditional ITO electrodes and various emerging ITO alternatives. Reproduced with permission [6]. Copyright 2019, Royal Society of Chemistry. (b) Representation of *FoM* for different TCEs plotted against *T_550nm_*, and *T_550nm_* values shown in relation to the inverse sheet resistance (1/*R_sheet_*) for various *FoM*, as well as for different widths (*w*) and pitches (*p*). Reproduced with permission [74]. Copyright 2014, John Wiley and Sons. (c) Diagram of TCE key parameters, (d) geometries. (e,f) Illustrate optoelectronic properties of the TCE grid line width and pitch. Reproduced with permission [6]. Copyright 2019, Royal Society of Chemistry.

Haacke's FoM values for various metal mesh TCEs can be visualized as a function of T_550nm_, as illustrated in Figure 2b. In addition to this approach, Dressel and Gruner introduced an alternative method for calculating the FoM that uses the ratio of direct current conductivity (σ_dc_) to optical conductivity (σ_opt_) at a specified wavelength, typically 550 nm [81]. This ratio (σ_dc_/σ_opt_) serves as the FoM and can be determined as follows:

(2) $$\frac{\sigma_{dc}}{\sigma_{opt}}=\frac{188.5}{R_{sheet\;}\left(T_{550\;nm}^{-\frac{1}{2}}-1\right)}\;$$

According to this formula, the visible‐range transmittance of a TCE is inversely related to its sheet resistance. A higher σ_dc_/σ_opt_ ratio indicates a better trade‐off between transparency and conductivity, meaning increased transmittance at a given sheet resistance, or reduced resistance for a set transmittance. Consequently, achieving a high *FoM* is crucial for TCEs to perform well in devices. For practical applications, a TCE should typically achieve a transmittance greater than 90% and a sheet resistance below 100 Ω/□, corresponding to a minimum *FoM* of around 35 [82, 83].

### 2.2 Unique Electrical and Optical Characteristics of TCEs

Increasing the aspect ratio (AR, thickness/line width) of mesh‐patterned TCEs improves both electronic and optical characteristics, such as conductance and transmittance. A high AR is achieved by increasing mesh‐line height or density, boosting conductivity while minimizing shadowing. Mesh geometry controls AR, and increasing AR (at a fixed width) lowers sheet resistance (R_sheet_), but excessive thickness increases surface roughness, which can hinder subsequent coating. Key design factors include the mesh's line width, pitch, web width, height, and surface type, as highlighted in Figure 2c. Equations by Van de Groep et al. relate *R_sheet_* to these features for square grids [84].

(3) $$R_{sheet}=\frac{\rho .p}{h.\omega }\;$$

The material's resistivity (*ρ*) and the mesh geometry strongly affect the sheet resistance (*R_sheet_*) of TCE electrodes. Because TCEs can have many patterns, their performance can't be compared by a single figure of merit. In square metal grids, a higher filling factor (*f_F_*) increases conductivity but also shadowing [85].

(4) $$f_{F}=\frac{2\rho w-w^{2}}{\rho^{2}}\;$$

Metal meshes block some light, causing shadowing (Figure 2d), and their transmittance (T) can be predicted from the filling factor (*f_F_*).

(5) $$T=1-\;f_{F}=\frac{{\left(\rho -w\right)}^{2}}{\rho^{2}}\;$$

Sheet resistance (*R_sheet_*) depends on grid width (w), pitch (ρ), and relates to transmittance (T) [74].

(6) $$R_{sheet,\;grid}=\frac{\rho }{h}\;\frac{\left(1-\sqrt{T}+T\right)}{\left(1-\sqrt{T}\right)}$$

Expressions (iii)‐(vi) apply only to square grid electrodes. Changing the mesh shape, such as switching from a hexagonal to a circular pattern, alters the aperture ratio and shadow loss and requires new calculations. Increasing *w* improves TCE conductivity but also increases the shadow area (Figure 2e), which is undesirable for optoelectronic devices like solar cells. A decrease in w leads to an increase in *R_sheet_*; however, this effect can be mitigated by employing thicker metal layers, as demonstrated in previous studies. When *p* is higher, light transmission improves, but *R_sheet_* also increases, and the reverse is true (Figure 2f). Excessively high *p* can reduce charge‐collection efficiency because carriers must travel longer diffusion paths. Both w and *p* should be optimized for conductivity and transparency. The web or window width affects the mesh's aspect ratio, and different fabrication methods produce varying widths. Mesh height (or thickness) also impacts performance: greater height lowers *R_sheet_* but raises surface roughness. Unlike the standard use of ITO or FTOs, the dimensions of these TCEs can be tailored to specific needs [6]. Key design points are: (i) keep metal line‐width below the wavelength for transparency, and (ii) use sub‐micrometer pitch for low resistance and even current distribution.

### 2.3 Unique Features and Stability

#### 2.3.1 Photonic and Electronic Scattering in TCEs

Most of the TCEs are composed of interconnected, regularly patterned metal lines that offer several benefits over conventional ITO or FTO electrodes. These structures can improve light scattering and coupling, and the degree of this enhancement depends on the nanoparticles' grain size within the mesh [86]. Consequently, TCEs are highly suitable for emerging electronic device applications, as increased light scattering lengthens the optical path of photons, increasing their likelihood of absorption in the active layer. Unlike ITO and metal nanowire electrodes, metal‐mesh TCEs generally do not generate haze, making them ideal for display and lighting applications where low haze is essential to maintaining high image clarity and color accuracy [87]. For instance, touch screen panel and transparent conductive heater applications on windshields or visors often require haze levels below 3% to preserve image sharpness. Furthermore, these TCEs can reduce electron scattering at grain boundaries or irregularities in metal‐film electrodes, thereby lowering lateral resistance, which leads to improved electrical conductivity [88].

#### 2.3.2 Adhesion Properties and Surface Conformity of TCEs

TCEs require strong adhesion to their substrates, whether rigid or flexible, to ensure effective integration into large‐area flexible optoelectronic devices. Adhesion can be evaluated through changes in sheet resistance (ΔR/R_0_) during adhesive tape tests [89]. For example, studies have shown that AgNW electrodes exhibit a significant increase in sheet resistance after only a few tape cycles, whereas Ag nano‐mesh electrodes maintain stable electrical properties. This suggests that metal‐mesh electrodes bond more securely to a range of substrate surfaces, including elastomeric materials, and are less prone to detachment compared to metal nanowires (MNWs) [90, 91]. Further research has demonstrated that strong adhesion between gold nano‐mesh networks and the substrate contributes to improved stretchability and durability through distributed rupture during elongation [92]. The periodic, interconnected design of mesh networks enables better substrate adhesion and greater flexibility than the random arrangement of MNWs. The robust adhesion characteristic further enhances the suitability of metal mesh electrodes for large‐scale printing methods.

#### 2.3.3 Mechanical Flexibility and Durability

The TCEs are being developed as alternatives to ITO for use in flexible and wearable optoelectronic devices. For these applications, TCEs must possess enough mechanical flexibility and durability to withstand repeated bending, twisting, folding, crumpling, and stretching. To assess mechanical robustness, most research studies employ bending tests as a primary benchmark. These tests typically measure two key variables: bending curvature, indicating flexibility, and the number of bending cycles, which relates to the material's fatigue strength. Performance is typically assessed by measuring the percentage change in electrode resistance (ΔR/R_0_), with ΔR representing the resistance change following deformation and R_0_ denoting the initial resistance. For example, a study demonstrated that integrating a silver grid with PEDOT:PSS to create a hybrid TCE achieved notable flexibility (bending radius around 1 mm) and sustained strong fatigue resistance (ΔR/R_0_ < 0.2%) after 10 000 bending cycles [93]. Similarly, Au nano‐meshes created using grain lithography, as reported by Guo et al., exhibited strong mechanical robustness with negligible ΔR/R_0_ or fatigue, even under 40% tensile strain [92]. Maurer et al. reported that nano‐imprinted gold nano‐meshes exhibited excellent mechanical durability [94]. Guo and Ren pointed out that relying solely on bending cycles does not fully capture TCE flexibility, as these tests produce only minor strains (approximately 5%) [95]. Stretching tests, which directly measure strain, along with other deformation assessments such as twitching tests, are also necessary for a more comprehensive and scientific evaluation of TCE performance [96].

#### 2.3.4 Chemical and Environmental Stability

This passage discusses the importance of chemical and environmental stability, particularly thermal and humidity resistance, for TCEs used in emerging electronic devices. Solution‐processed electrodes (such as Ag and Cu NWs) are more prone to degradation, whereas TCEs, particularly gold, offer superior stability due to their greater resistance to oxidation [95]. Comparative studies show that materials and design choices significantly affect the stability of TCEs under heat, humidity, and chemical exposure [87]. Gold mesh electrodes are the most stable, silver is moderately stable, and copper is the least stable unless protected by coatings [6, 97, 98]. The text concludes by emphasizing the need for future research to enhance the durability and sustainability of mesh‐based electrodes for long‐term outdoor use.

### 2.4 Material‐Based Categories of TCEs

This section explores the various types of TCEs, highlighting their structures, opto‐electronic properties, and applications. Key categories include metals (Au, Ag, Cu, etc.), metal oxides, conductive polymers, carbon‐based materials, and composite electrodes [99]. Understanding the distinctions among these types is crucial for selecting suitable materials for emerging electronics like PVs, PDs, LEDs, touchscreens, and energy storage devices.

#### 2.4.1 Metal Mesh‐Based TCE

These electrodes are often constructed from extremely thin metal layers, such as silver, gold, or copper, or from structures such as metal nanowires and grids. Their properties can be tailored by incorporating materials such as oxides or specialized polymers. It can be divided into metal nanowires (NWs), films, and grid‐like structures. Initially, Ag NWs produced via solution processing achieved high optical transmittance (96.5% at 450 nm, as shown in Figure 3a) and low *R_shee_*_t_ (11.7 Ω/□, as shown in Figure 3b), and have been employed as TCE in inorganic III‐nitride LEDs [33]. To improve the TCE performances, the metals or metal NWs have been patterned or printed over the rigid and flexible substrates. Metal‐mesh‐type TCE has emerged as the top‐most promising alternative to conventional ITO for flexible optoelectronic devices [74]. Unlike brittle continuous oxide films, the metal mesh electrode uses highly conductive metal paths arranged in a micro‐scale grid pattern, allowing electrical current to flow through the metal lines while incident light passes through the openings. Figure 3c shows the flexible OLED device fabricated using the Ag grid/PEDOT based TCE as the bottom electrode, showing good luminance efficiency at a high luminance of 10k cd/m^2^. The digital image of the fabricated device is presented in the inset of Figure 3d, which also shows a stable power efficiency at high luminance 10k cd/m, showing the excellent ability of the TCE. This structural concept enables metal mesh electrodes to effectively mitigate the conductivity‐transparency trade‐off that often limits conventional continuous transparent conductive films. Its attractiveness stems from the high electrical conductivity of metals and the ability to independently adjust optical transparency and R_sheet_ through geometric design. In another attempt to address these limitations (high process temperatures and the brittle nature of TCO), Rahman et al. utilized Cabrera‐Mott oxidation to create large‐area, two‐dimensional (2D) TCE via liquid metal printing [100]. The process is depicted in Figure 3e. This robotic, vacuum‐free method enables the deposition of ultrathin (2–10 nm) indium tin oxide (ITO) films that offer excellent flexibility, transparency (>95%), and conductivity (>1300 S/cm), achieved by printing with hypoeutectic In‐Sn alloys at temperatures below 140°C. The thickness variation of the ITO layer with printing speed is presented in Figure 3f, with Figure 3g showing the histogram of the thickness of 1‐, 2‐, and 3‐layer ITO. Zhao et al. fabricated TCE using Ag NWs, utilizing a scalable inkjet printing method to pattern all PeLED device layers from the bottom anode to the top cathode [101]. The *R_sheet_* and T_500 nm_ of the inkjet‐printed AgNW‐based TCE show 9.56 Ω/□ and 64.5%. Ag and Cu mesh electrodes are used for their high conductivity and transparency. Ag meshes offer stability and flexibility but are expensive. Metal‐mesh electrodes are more durable than oxides and recent designs improve compatibility with thin layers [29, 36]. Ag mesh/ITO composites perform well in flexible OLEDs, but cost limits Ag's use. Cu mesh electrodes are a cheaper alternative to Ag, with good conductivity and transparency, especially when coated with oxides. However, they are prone to oxidation and need protection for stable use. Hybrid meshes with metals like Au and Ni address the limits of single materials. Au adds stability as a thin layer, while Ni improves adhesion and strength. Choosing mesh materials involves balancing conductivity, cost, stability, and device needs. Metal mesh TCEs can suffer from haze, visible patterns, and roughness, affecting device performance.

**FIGURE 3:**
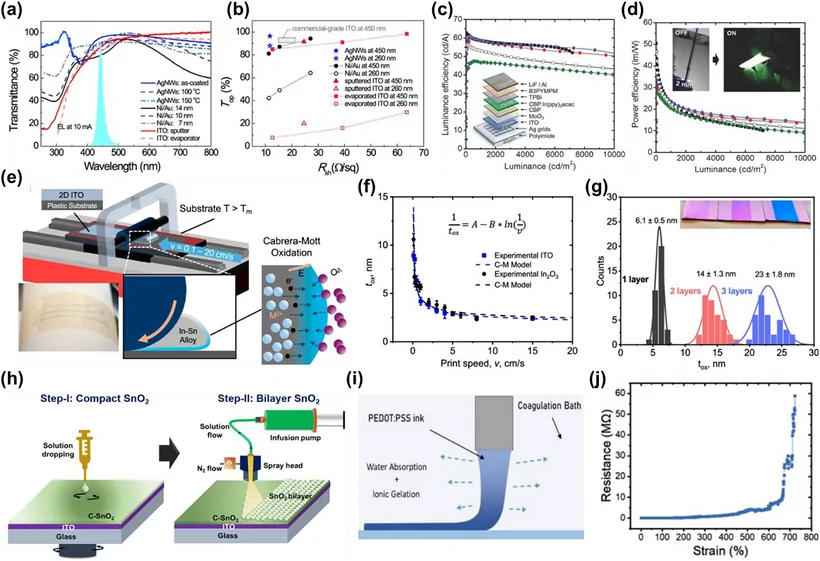
(a) Transmittance and (b) Transmittance vs sheet resistance variation of AgNW, different‐layer Ni/Au and physically deposited ITO‐based TCE, Reproduced with permission [33]. Copyright 2015, Creative Commons Attribution 4.0 International License; (c) Luminance efficiency of Ag patterned/ITO TCE‐based flexible OLED device, (d) Power efficiency and inset showing the digital image of the flexible OLED, Reproduced with permission [74]. Copyright 2013, John Wiley and Sons; (e) Schematic illustration of the In‐Sn liquid‐metal printing, (f) Thickness vs. printing speed, (g) Thickness vs ITO layer, (inset) appearance of the actual films. Reproduced with permission [100]. Copyright 2024, Creative Commons Attribution‐NonCommercial‐NoDerivatives 4.0 International License, (h) Schematic illustration of the SnO_2_ spray coating process. Reproduced with permission [102]. Copyright 2023, Royal Society of Chemistry; (i) Illustration of the wet‐printing process and (j) resistance of the printed PEDOT‐based TCE with increasing stretching. Reproduced with permission [103]. Copyright 2024, John Wiley and Sons.

Metal‐based TCEs are usually the go‐to materials where a very low sheet resistance is required owing to their high intrinsic conductivity. However, the conductivity of random AgNW‐based TCEs depends not only on the resistance of individual nanowires but also on the fabrication process and on effective junction contact between wires, which can lead to lower junction resistance, junction displacement, or junction failure under repeated bending cycles. Additionally, they are susceptible to environmental and electrochemical corrosion and electromigration under prolonged use, further limiting their commercial use. Despite their high conductivity, the performance of AgNW‐based TCEs largely depends on nanowire–nanowire junction resistance, and repeated deformation can cause junction separation, nanowire sliding, or fracture, progressively increasing sheet resistance. Further, their increased surface roughness and protruding nature induce leakage currents and short circuits in ultrathin devices, while also having weaker adhesion to polymeric substrates reduces mechanical durability. Additionally, AgNWs suffer from oxidation by atmospheric oxygen, sulphidation, corrosion, and electromigration. Strategies to improve junction welding, embedding to improve adhesion and surface smoothness, encapsulation to suppress corrosion, and core–shell structures to enhance stability are among the most widely applied methods to improve AgNW performance. Hence, for demanding flexible optoelectronic applications, the most reliable strategy is to combine one or two of these techniques. Future studies should assess such electrodes in coupled accelerated‐aging protocols that evaluate spatial and temporal trends in sheet resistance under cyclic strain, elevated temperature and humidity, continuous electrical bias, sulfur‐ or halide‐containing environments, and realistic device operation. Such reliability‐oriented assessment, not just initial conductivity and transparency, will be the ultimate factor determining whether AgNW TCEs can compete with ITO in large‐area commercial electronics [104].

Metal meshes, while reducing some AgNW‐related problems, introduce issues such as geometric shadowing, visible patterns, topographical roughness, and non‐uniform current distribution at higher pitches. Cu mesh, while being a suitable, cheaper replacement for Ag, still needs oxidation resistance, while Au provides higher stability but at a significantly higher cost. Overall, metal‐based TCEs are promising, with future studies focusing on addressing their shortcomings.

#### 2.4.2 Metal Oxides‐Based TCEs

Metal oxide‐based transparent electrodes, especially oxide‐TCEs, have long served as the dominant electrode platform in emerging electronics due to their high optical transparency, low electrical resistance, and excellent compatibility with existing thin film device architectures. Among these materials, ITO remains the most commonly used because it offers a strong combination of high visible‐light transparency and electrical conductivity. In addition to ITO, other oxide electrodes such as indium zinc oxide (IZO), aluminum‐doped zinc oxide (AZO), gallium‐doped zinc oxide (GZO), and fluorine‐doped tin oxide (FTO) have also been widely studied as alternatives or supplements depending on the structure and manufacturing process of the target device. Metal oxide electrodes have smooth, continuous films that help deposit thin layers in devices like LEDs and solar cells [105]. Their low roughness prevents shorting and ensures uniform charge flow. With low haze and high clarity, they are the standard for transparent electrodes in displays and photovoltaics [65, 106]. However, metal oxide electrodes like ITO are brittle and crack under bending, making them less suitable for flexible devices. Some require high‐temperature or vacuum processing, which isnot ideal for plastics or cheap manufacturing. Scarcity and cost of indium also drive the search for alternatives. To solve these issues, research explores low‐temperature processes, amorphous oxides, ultrathin films, and multilayer oxide/metal/oxide (OMO) structures [106]. Amorphous oxides like IZO are more flexible than crystalline ITO. ZnO‐based electrodes reduce indium use and offer some flexibility, though with lower conductivity. OMO structures combine oxide smoothness with metal flexibility and conductivity. Liu et al., fabricated TCE by blade‐coating a hydrocolloid of SnO_2_ along with an aqueous dispersion of AgNWs [107]. This process resulted in a composite AgNWs:SnO_2_ film with a uniformly smooth surface, where the AgNW network is effectively encapsulated by the SnO_2_ colloid. The resulting transparent film exhibits a *R_sheet_* of approximately 17 Ω/□ and a high transmittance of about 92% at 550 nm. In our group, we have developed SnO_2_ as ETLs for PSCs using spray printing with more than 93% optical transmittance [102]. The spray coating process is depicted in Figure 3h.

ITO is still the benchmark for TCEs owing to its sheet resistance, high visible transparency, low haze, excellent surface smoothness, and established industrial processing. ITO as a TCE is suited to a variety of devices containing nanoscale functional layers such as OLEDs, photovoltaics, and other vertically stacked devices. However, the ceramic nature of ITO promotes crack formation during repeated bending or tensile strain, leading to an irreversible increase in resistance. Also, its dependency on indium and the requirement of vacuum deposition techniques, and sometimes high operating temperatures, often present cost and sustainability concerns. The other oxides like AZO, GZO, IZO, and FTO often reduce these constraints, but can compromise on conductivity, stability, and mechanical flexibility. Thus, the oxide‐based TCEs remain mainly suitable for rigid and moderately flexible platforms where highly smooth interfaces are required, and the use of metallic or other TCEs is limited. Further, research should focus on the reduction of indium composition, low‐temperature fabrication processes, improved strain tolerance, and strategies for recycling and recovering these oxides.

#### 2.4.3 Conducting Polymer‐Based TCEs

Conducting polymer‐based TCEs have attracted significant attention as suitable candidates for flexible optoelectronics, as they combine conductivity with intrinsic mechanical softness, low‐temperature processing, and solution‐based manufacturing. Unlike brittle oxide electrodes, conducting polymers can be deposited on plastic or elastomeric substrates through coating and printing processes, making them particularly suitable for lightweight, deformable, and potentially low‐cost devices [64, 75, 108]. Poly(3,4‐ethylenedioxythiophene):poly(styrenesulfonate) (PEDOT:PSS) stands out as one of the most thoroughly studied and widely used conducting polymers due to its unique properties. PEDOT:PSS is valued as a transparent electrode for its high transparency, tunable work function, good stability, and compatibility with solution processing. Its flexibility suits polymer substrates better than oxides, improving bending and durability [75, 109, 110, 111]. However, its lower conductivity compared to metals or oxides limits its use in applications needing very low resistance. Excess PSS also causes moisture issues and reduces stability. To address this, methods like doping and solvent or acid treatment are used to boost conductivity while keeping transparency and flexibility. PEDOT:PSS is also versatile for hybrid electrodes. It's often combined with metals, nanowires, carbon, or oxide layers to improve conductivity while keeping flexibility. These hybrids can enhance adhesion, charge collection, work‐function matching, and stability, making conducting polymers valuable components in advanced transparent electrodes. Recently, Zhang and co‐workers introduced a new wet‐printing method for creating highly stretchable PEDOT:PSS micro‐TCE. This technique uses a liquid coagulation bath, making the process non‐contact, simple, rapid, and ideal for printing inks with low viscosity [103]. The schematic illustration of the process is presented in Figure 3i. The method enables the formation of PEDOT:PSS lines as narrow as 20 µm. Incorporating D‐sorbitol as a plasticizer results in PEDOT:PSS electrodes that maintain conductivity and minimal strain sensitivity even when stretched beyond 720% (as shown in Figure 3j).

PEDOT:PSS is a very versatile material for the fabrication of TCEs owing to its intrinsic flexibility, tunable work function, low‐temperature processability, and capability of large‐area printing. However, pristine PEDOT:PSS has higher sheet resistance than ITO or metallic TCEs and requires the aid of secondary dopants to alter its conductivity, mechanical and surface properties, and long‐term stability. Apart from this, the PSS phase is acidic, which promotes degradation of the adjacent layers and corrosion of electrodes, and is also hygroscopic in nature, which affects the film integrity and conductivity. The loss of dopant, phase segregation, and changes in film organization of PEDOT:PSS leading to increased resistance during humidity and thermal exposure, further impairing its use. Hence, PEDOT:PSS has clear advantages in flexible, low‐temperature printable electronics but has limited applications as a standalone electrode, and in low‐resistance and long‐term operations. Further research on strategies for low PSS or PSS‐free conducting polymers is needed and needs to be combined with metal meshes and other oxide‐based materials to improve conductivity, adhesion, and effective interfacial charge injection.

#### 2.4.4 Carbon‐Based Materials for TCEs

Carbon‐based materials have become attractive alternatives to traditional TCEs, such as ITO and FTO, owing to their superior electrical conductivity, high optical transparency, chemical and corrosion stability, and inherent mechanical stability [112]. For next‐generation flexible and optoelectronics applications, research has focused on carbon‐based materials such as Carbon nanotubes and graphene because of ITO's brittleness, high cost, and scalability limitations [63]. Among carbonaceous materials, Carbon nanotubes (CNTs) have attracted recent attention as a result of their superior surface area, porosity, high mechanical and thermal stability, and excellent photoelectric properties. CNTs are divided into single‐walled (SWCNTs) and multi‐walled (MWCNTs) carbon nanotubes based on the total number of carbon layers, with SWCNTs having higher conductivity. Zhang et al. fabricated a SWCNT film by growing it using a ferrocene‐based FCCVD method [113]. They doped the films with PTA by dipping them in an aqueous PTA solution (100 mg/mL), then transferring them onto glass or silicon substrates. These PTA‐doped SWCNT films had a lower sheet resistance of 60 Ω/□ at 90% optical transmission, which was 46% ± 10% lower than that of pristine SWCNT films, while still maintaining excellent mechanical flexibility and long‐term stability at ambient conditions. Khabushev et al. studied the correlation between the effects of nanotube structural parameters on the optoelectronic properties of the single‐walled CNT film [114]. In the study, SWCNTs were fabricated via CO‐based aerosol CVD using a Fe‐based catalyst. The study found that lower defect levels in CNTs led to higher conductivity in thin films. A pristine SWCNT film with an I_G_/I_D_ ratio of 330 achieved a sheet resistance of 430 Ω/□, the lowest among all, while exhibiting a 90% optical transmittance. In another study, Boyd et al. directly compared free‐standing thin films of SWCNTs and MWCNTs fabricated from chloro‐sulfonic acid dispersions [115]. The first RF study of free‐standing CNT thin electrodes, was achieved by measuring the RF reflectance, transmittance, and shielding efficacy of the SWCNT and MWCNT films using a rectangular waveguide from 10 to 15 GHz. As SWCNT film thickness increased, a drop in optical transmittance and an increase in RF reflectance were observed. The advantage of these free‐standing CNT films as TCEs was demonstrated by 60% RF reflectance along with an optical transmittance of ≥ 50%. Zhang et al. fabricated CNTs with high aspect ratio via the FCCVD method using a ferrocene (FeCp2) catalyst and methane (CH_4_) as the carbon source for application as TCFs in optoelectronics [76]. The synthesized high‐aspect‐ratio CNTs facilitate the formation of a continuous network of DWNTs during dry filtration, leading to DWNT films that can be further transferred onto glass and PET substrates to act as TCFs. The TCF containing DWNTs with a of ∼4 nm diameter (Figure 4a) exhibited a low sheet resistance of 35 Ω/□ (Figure 4c) at 90% optical transmittance, and a PSC (Figure 4b), fabricated with it had a power conversion efficiency (PCE) of 17.4%. Koo et al. developed a highly durable and flexible graphene TCE, developed by directly spin‐coating polyimide (PI) onto CVD‐grown graphene on a Cu substrate [116]. Figure 4d shows the fabrication process of the TCE. In the process, PI acted as both substrate and carrier for the graphene, and the PI@Gr TCE had a high transmittance of 92% at a sheet resistance of 83 Ω/□. Due to the improved connection between graphene and PI, a solar cell fabricated with these PI@GR TCEs exhibited a PCE of 15.2% along with excellent mechanical robustness, as is evident from the repeated bending study at a radius of 2 mm in Figure 4e. In another study, Kim et al. explored the Pt doping of graphene through selective atomic layer deposition (ALD) to enhance its electrical conductivity [117]. Pt ALD led to a reduction in sheet resistance from 471 to 86.8 Ω/□, while still maintaining an optical transmittance of 90% at 550 nm after 200 cycles. Subsequently, the Pt ALD was further used to fabricate a multi‐layer graphene film, resulting in a sheet resistance of 75.8 Ω/□ while maintaining a high transparency of 87.3% after 200 cycles. Yao et al. fabricated a rGO‐based TCF through spray coating of 0.2 mg/mL GO solution on a pre‐heated glass substrate under an HI reduction atmosphere [118]. The study concluded that using 0.2 mL of HI, the flexible rGO/PET exhibits sheet resistance below 1000 Ω/□ and a transparency of 80%. While using 0.03 mL HI, the rGO/CC‐based SC exhibited a high gravimetric capacitance (C_sp_) of 433.2 F/g at a scan rate of 5 mV/s, with a capacitive retention of 93.26% after 20 000 GCD cycles. Choi et al. prepared fullerene (C_60_)/Ag/C_60_ (CAC) multilayer TCF via multilayer thermal evaporation [119]. The CAC TCF thus fabricated exhibited a low resistance of 5.63 Ω/□ and transmittance of 66.13% at 550 nm. The thermally evaporated CAC exhibited a PCE of 5.1% for photovoltaic solar cells with an active area of 1.08 cm^2^ and also achieved a saturation temperature of 116.6°C at a low output voltage of 4.5 V when applied as a thin‐film heater. In our lab, Ovhal et al. used 3D printing with a commercial carbon black paste to fabricate carbon/Ag/Carbon‐based TCE (Figure 4f) for application in a sandwich‐type transparent all‐solid‐state supercapacitor [108]. In the study, Ag‐embedded carbon black grid electrodes were 3D printed on both sides of a dialysis membrane, which served as the separator. Figure 4f shows how the line pitch between the electrodes was controlled to achieve the highest optical transmittance of 82% at 2.1 mm, up from 52% at 0.6 mm, while maintaining good conductivity. The variation of surface area with thickness of electrodes and the subsequent variation of transmittance with surface area depicted in Figure 4g also represents that with increasing thickness of the electrode lines and the line pitch of the TCE, the transmittance gradually increases to ∼90% while maintaining similar sheet resistance. Overall, carbonaceous materials such as CNTs, graphene, and others possess strong potential as alternatives to traditional TCEs owing to their high transparency, good conductivity, and mechanical durability. Carbon‐based TCEs are excellent for flexible electronics requiring high chemical resistance and mechanical deformability but are limited in their electrical performance compared to other materials. In the case of CNTs, the charge transfer process is heavily influenced by nanotube length, chirality, defects, and tube‐tube junction resistance. Also, increasing CNT density in order to obtain higher conductivity heavily impacts the transparency of the electrode and increases haze. In the case of graphene, although it has great charge mobility and only absorbs a lower amount of visible light per layer, it still possesses an equivalently low sheet resistance. Also, during CVD growth and consequent transfer, cracks, wrinkles, and structural defects increase the resistance of the TCE. Hence, CNT‐ and graphene‐based TCEs are more suitable for low mass, flexible, and highly chemically durable applications than for ultralow sheet resistance applications. The most promising approaches toward practical carbon‐based TCEs are transfer‐free growth of graphene, cleaner large‐area transfer processes, stable molecular doping, controlled chirality and dispersion of CNTs, junction engineering and hybridization with metallic or polymeric conductors.

**FIGURE 4:**
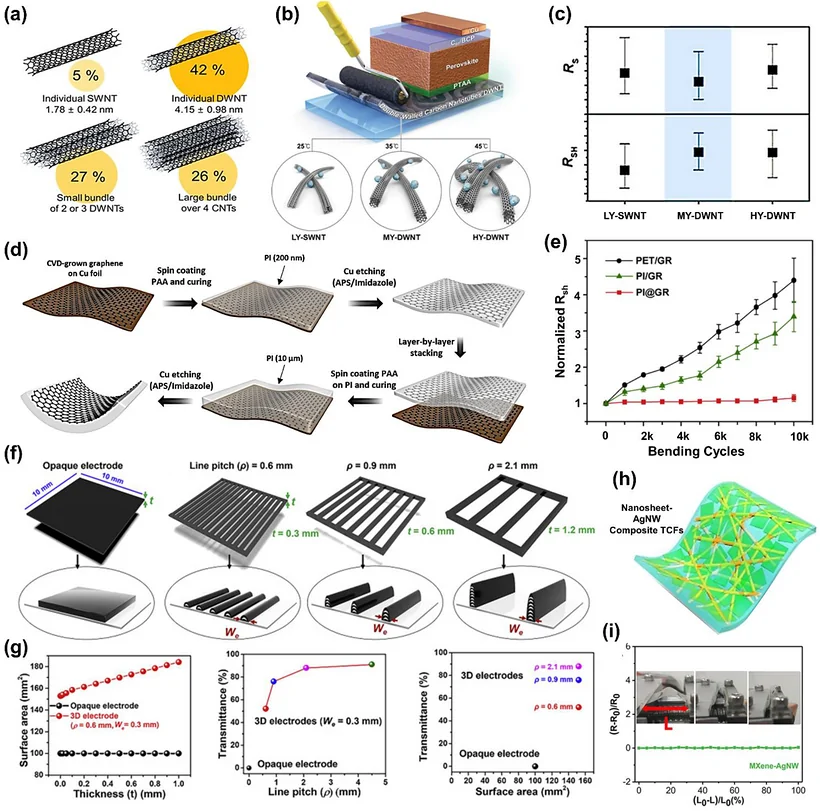
(a) Application of CNT (3D illustration), TCF‐based PSCs, and examination of MY‐DWNT TCF's components. (b) Proportional distribution for the MY‐DWNT TCF with comparison of the CNT‐based PSCs according to (c) R_S_ (above) and R_SH_ (below). Reproduced with permission [76]. Copyright 2022, John Wiley and Sons. (d) Schematic illustrating the fabrication process of PI@GR. (e) Variation in normalized *R_sheet_* of PET/ITO, PET/GR, PI/GR, and PI@GR over 10K bending cycles at 5 mm bending radius. Reproduced with permission [116]. Copyright 2020, Elsevier. (f) Multi‐layer printed fiber‐shaped 3D‐SC with 5‐layers of MAM‐electrodes. (g) Electrical and optical characteristics of 5‐layered MAM‐ 3D electrodes. Reproduced with permission [108]. Copyright 2021, Elsevier. (h) Schematic of nanosheets‐AgNW composite TCF. (i) Relative resistance (R‐R_0_/R_0_) with change in length (L_0_‐L/L_0_). Reproduced with permission [77]. Copyright 2021, Elsevier.

#### 2.4.5 Composite‐Based Materials for TCEs

Carbon‐based TCEs and TCFs exhibit good chemical resistance but often exhibit higher resistance and lower optical transmittance. To obtain low sheet resistance and high transparency, a composite of metal‐based and other materials, including carbonaceous materials and conducting polymers, is often used. Jin et al. from our laboratory fabricated a metal‐embedded TCE (ME‐TCE) by gravure‐printing Ag strips over spin‐coated PEDOT:PSS on a glass substrate, followed by NOA63 encapsulation and UV curing [120]. Fabricated ME‐TCEs were detached from the glass substrate and, with an electrode gap of ∼190 µm, exhibited a transmittance of ∼63%, which reduced to ∼49% for an electrode gap of ∼90 µm. The ME‐TCE with the larger electrode gap was used for OSC applications due to its higher light absorption and reduced shadow power loss, enabling the SF‐OSCs to achieve a PCE of ∼6.43%. Additionally, the ME‐TCE also enabled SF‐SCs to exhibit a Ca of ∼52 mF cm^−2^ with a capacitance retention of ∼96% after 5000 GCD cycles. A Ti_3_C_2_Tx MXene TCE was fabricated by Kumar et al. via spin coating onto a PET film, followed by blade coating of active material for electrochromic application [121]. The MXene‐based flexible TCE exhibited a sheet resistance of ∼424 Ω/□ and an optical transmittance of ∼87%, and was stable under multiple bending cycles. With a high transmittance of almost 80% at 60 V and a low threshold voltage of less than 10 V, this TCE exhibited excellent transparency in the ON state and decent opacity in the OFF state when applied to smart windows. Bai et al. fabricated MXene–Ag NW TCFs via solution processing (Figure 4h), yielding a resistance of 16 Ω/□ and an optical transmittance of 86.1% [77]. Further, the TCE used in EMI shielding application had an EMI shielding effectiveness (SE) of 27.8 dB at 8–13 GHz, and, as Figure 4i shows, it maintained its optoelectrical properties even after 5000 bending cycles and surviving 90 s of corrosion in a Na_2_S solution, demonstrating the excellent stability of the TCE. Kang et al. fabricated Cu/graphene hybrid films via CVD that function as both p‐type and n‐type TCE in both traditional and inverted OSCs [122]. 7 nm Cu‐graphene exhibited a sheet resistance of ∼75 Ω/□ with 85% transmittance, indicating good optoelectronic properties. Nair et al. developed a stable nanocomposite ink using Ag NWs and PEDOT: PSS and fabricated TCEs via the direct ink writing technique [123]. This TCF had 86% transparency and at resistance of 23 Ω/□ and was used in two applications: a flexible transparent CPW‐fed monopole antenna and a flexible transparent heater. The antenna exhibited performance similar to commercially available Wi‐Fi antennas, also having added benefits of flexibility and transparency. A 50 × 20 mm transparent heater using this TCE showed a maximum saturation temperature of 85°C at an input voltage of 6 V. Wan et al. reported a solution‐processed flexible OSC using PEDOT:PSS anode doped with trifluoromethanesulfonic acid (CF_3_SO_3_H) as the TCE having sheet resistance of 35 Ω/□ [124]. These flexible OSCs achieved a PCE of 16.41% and maintained stable performance over 1000 bending cycles (at 1.5 mm radius) and at 85°C temperature, demonstrating high mechanical robustness with good thermal stability.

MXenes (especially Ti_3_C_2_Tx) inspite of having high metallic conductivity, hydrophilicity, good surface tunability, and mechanical durability, are limited to applications in printed TCEs due to their easy oxidizing nature because of exposure of ambient oxygen, moisture, and temperature. Another issue is the restacking tendency of MXene sheets, leading to charge transport loss and altered optical properties, while the variation in flake size and surface functionalization limits the reproducibility of conductivity and work function. Hence, the use of antioxidant additives, surface passivation, stable surface termination, and surface engineering are important further directions of research for MXenes. Composite TCEs/TCFs (e.g., AgNWs or metal meshes mixed with graphene/CNTs, conductive polymers, oxides, or 2D materials) offer a viable path to optoelectronic electrodes that balance high transparency, low sheet resistance, and low haze while improving adhesion and surface smoothness. These hybrids can outperform single‐component films in OLEDs, solar cells, touch panels, and transparent heaters, offering improved mechanical reliability by leveraging complementary roles: conductive backbones for percolation and protective/planarizing layers for stability. However, hybridization is not inherently superior. Additional interfaces can introduce contact resistance, thermal‐expansion mismatch, possible galvanic or chemical reactions, interlayer delamination, increased processing complexity, and greater difficulties in recycling and compositional reproducibility. Hence, the future research direction is not only combine the different materials but also design multifunctional interfaces that address failure mechanisms while maintaining good conductivity and optical properties and keeping manufacturing complexity low.

In all, there is no single TCE platform that addresses all the factors like conductivity, optical transparency, mechanical deformability, environmental stability, processability, and cost‐effectiveness together. For instance, ITO has high transparency, low haze, and smoothness but is brittle, requires vacuum processing, and depends on indium. AgNWs offer better flexibility and good solution processability but have a rough surface and wire‐junction resistance. Metal meshes show lower sheet resistance and better current collection than nanowires, their performance is highly dependent on the linewidth, pitch, thickness, and pattern uniformity and also has limitations like optical shadowing. PEDOT:PSS have excellent flexibility and printability but possesses lower conductivity and has acidity and hygroscopicity and low environmental stability. CNT films have excellent mechanical robustness but suffer from nanotube junction resistance, bundling, and the conductivity‐transparency trade‐off. Graphene offers excellent intrinsic carrier transport, transparency, and flexibility, but practical films are limited by relatively high sheet resistance, grain boundaries, wrinkles, cracks, and unstable chemical doping. MXenes possess high conductivity and excellent solution processability but suffer from severe oxidation and environmental stability problems. Therefore, hybrid TCEs are increasingly the best engineering compromise, with metallic pathways providing low resistance, and polymers, carbon materials, oxides, or MXenes providing planar surfaces, flexibility, adhesion, interfacial control, or protection. Thus, the best choice of electrode depends on the failure modes relevant for the given application and not just on the lowest sheet resistance or the highest optical transmittance.

## 3 Techniques for Fabrication of TCEs

The high‐quality TCEs via solution processing are challenging because metal networks require precise uniformity and pattern alignment. Different printing technologies (Figure 5) have become popular for large‐scale TCE production because they are straightforward, compatible with roll‐to‐roll manufacturing, and do not require a vacuum. As a result, printing methods are less time‐ and cost‐intensive than traditional physical and chemical approaches. However, when designing with directly printed TCEs on rigid substrates, it is essential to consider thickness and surface roughness, especially for emerging electronic devices that use thin functional layers only a few hundred nanometers thick. Overlooking these factors may lead to electrical short circuits between the metal mesh and the top electrode in emerging electronic devices. To mitigate this risk, strategies include adding an additional conductive passivation layer to the TCEs or embedding the TCEs in a plastic elastomeric substrate, with or without an additional transparent electrode layer. Both solutions can produce hybrid TCEs with enhanced optoelectronic characteristics. Combining a mesh‐type electrode with a TCE (creating a hybrid TCE) usually results in better conductivity and device performance than using only a mesh‐type electrode.

**FIGURE 5:**
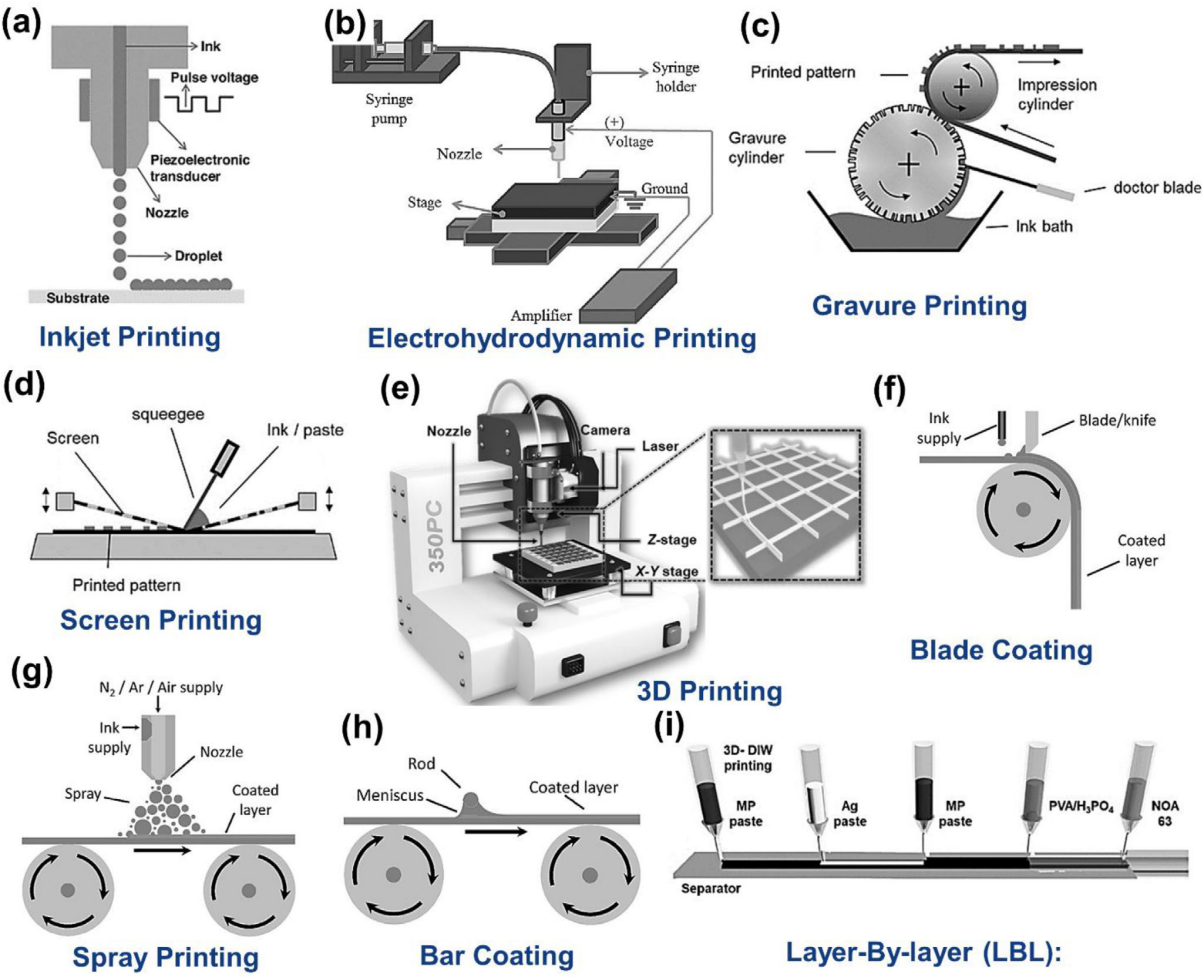
Schematics of the various printing techniques used for the fabrication of TCEs: (a) Inkjet Printing, (b) Electrodynamic printing, (c) Gravure printing, (d) screen printing, (e) 3D printing, (f) blade coating, (g) bar coating, (h) Spray printing, and (i) layer‐by‐layer printing.

### 3.1 Inkjet Printed TCEs

Inkjet printing offers an efficient approach for large‐scale manufacturing with minimal material waste. The drop‐on‐demand (DOD) method maximizes material usage, which helps limit the disposal of hazardous substances. In addition, DOD inkjet printing provides accurate digital management of ink droplets, allowing direct pattern creation and presenting valuable opportunities for the additive production of electronic devices and integrated circuits. Moreover, because it does not require contact or masks, the fabrication process is simplified and more accessible [125]. A further advantage of this technology is its ease of film deposition on curved surfaces and its suitability for rough or non‐flat substrates. A more recent development is the use of inkjet printers to deposit various inks onto substrates; this process enables the practical implementation of scalable, cost‐effective, and environmentally friendly processes [126, 127]. In terms of operation, inkjet printing functions as a flexible and non‐contact method for depositing functional layers with liquid‐phase inks, whether they are homogeneous or colloidal [128, 129, 130]. In inkjet printing, the piezoelectric transducer produces a pressure pulse that pushes the precursor ink through the nozzle (Figure 5a). A variety of inkjet systems have different mechanisms of droplet formation. The continuous ink jetting (CIJ) operating process involves applying pressure to an ink reservoir to maintain a constant flow of ink, thereby separating the ink into droplets. Its main advantage lies in the high frequency of droplet generation and printing. However, this technology is generally employed for the fabrication of large amounts and low‐quality applications. In the DOD system, droplets are produced exclusively on demand, thereby enabling considerable material savings compared to the CIJ system. The ink droplets are generated by the pressurized ink‐supply pump, which forces ink out of the nozzle. This step is typically modulated by a piezoelectric or thermal transducer. Piezoelectric DOD inkjet technology is increasingly used in industrial processes, as it supports inks with a wide range of viscosities and enables high printhead integration density. Implementing DOD inkjet printing requires addressing three main challenges: (i) Steady jetting of the ink; (ii) The interaction between the droplet and substrate begins once the droplet lands on the surface; and (iii) Wet film drying and annealing, which determine the final thin‐film shape and morphology. All variables associated with solutes affect the preparation of printed patterns, making ink development vital. Essential factors to consider are the ink viscosity, surface tension, and nozzle diameter. Mostly, the TCE is fabricated using inks of Au, Ag, AgNWs, PEDOT:PSS, SWCNT, graphene, and ITO NPs [127, 131, 132, 133, 134, 135, 136, 137, 138, 139, 140]. AgNW patterns as narrow as 10 µm can be made by selective deposition of poly(methyl methacrylate) layers with an inkjet printer. Custom designs, including logos and text, can be created on A4 samples and transferred to various substrates like silicon wafers, curved glass, or beakers [141]. Recently, Jena et al. developed TCE by molecular bridging with PEDOT:PSS, which enhances AgNW adhesion to PET and anchors Ti_3_C_2_T_x_ nanodots, forming efficient electronic connections and reducing resistance [142]. The resulting TCEs show 90.6% T_550_ and 10.3 Ω/□ R_sheet_, retaining 74.5% T_550_ and 11.1 Ω/□ R_sheet_ after 10 000 bends.

Overall, inkjet printing is appealing for maskless digital patterning, rapid design modification, lower material wastage, and direct deposition on different kinds of substrates. However, it is limited by a narrow printable rheological window, with parameters like viscosity, surface tension, particle size, evaporation rate, and substrate wettability that need to be carefully controlled for stable droplet formation. Problems like nozzle clogging, satellite drop formation, coffee‐ring formation, and particle aggregation all result in variations in sheet resistance. These issues are often compounded by concentrated AgNW, CNT, graphene, or MXene inks. Future progress requires suitable ink design solving the issues of clogging, waveform optimization, new drying technologies, and in‐line defect detection to preserve uniformity while maintaining transparency.

### 3.2 Electrohydrodynamic Jet Printed TCEs

Electrohydrodynamic (EHD) jet printing offers an affordable, contactless, and efficient method to directly produce high‐resolution patterns used in both micro‐ and nanofabrication [143]. EHD jet printing is an advanced inkjet technique that creates ultra‐fine ink droplets using an electric field between the nozzle and substrate (Figure 5b). This approach enables high‐resolution direct printing, which is crucial in the fabrication of a wide range of devices [143, 144, 145]. Importantly, the performance of EHD jet printing is easily tuned by altering the electric field strength, and the process supports a diverse range of functional inks. This technique attracts substantial research attention due to its exceptional features such as sub‐micron resolution (less than 1 µm), broad ink viscosity range (1–10 000 cps), adjustable printing modes, and suitability for flexible or wearable technologies [145, 146, 147, 148, 149]. In EHD jet printing, material deposition is achieved through the use of an electric field, setting it apart from standard inkjet printers. By applying voltage to the nozzle tip, a Taylor cone is generated, enabling the ejection of ink droplets or jets significantly smaller than the nozzle diameter, rather than relying on high pressure [150]. This process creates nano‐ and micrometer‐scale patterns that conventional inkjet methods cannot achieve, as those require impractically high pressures for small nozzles. The electric field gathers charged particles at the meniscus, and once the voltage is high enough, the surface tension is overcome, and fine jets are produced [144]. The EHD jet can be used to print several types of electrodes using inks such as metals, metal oxides, carbon materials, conductive polymers, and composites.

EHD extends DIW printing to considerably lower feature sizes, making it highly efficient for ultrafine transparent metal grids with narrow conductive lines, eventually minimizing optical shadowing. However, stable printing is often dependent on a variety of factors like electric‐field intensity, nozzle‐substrate distance, ink conductivity, viscosity, and environmental conditions. Hence, with a tiny variation in any of these parameters can lead to nonuniform line widths through the destabilization of the Taylor cone. Additionally, the use of high electric fields restricts the choice of inks and substrates. To translate its laboratory‐scale resolution advantage to scalable TCE manufacturing, multi‐nozzle systems, closed‐loop electric‐field control, standardized ink‐property windows, and high‐speed positioning must be optimized.

### 3.3 Gravure and Gravure Offset Printed TCEs

Gravure printing is one of the high‐throughput roll‐to‐roll printing technologies valued for its low per‐unit cost, excellent scalability, and continuous production capability. It operates by transferring ink from engraved microcells on a rotating gravure cylinder: the cells are filled with ink, a doctor blade removes excess, and the ink is transferred onto the substrate under controlled pressure, followed by drying/annealing to form the desired pattern (Figure 5c). It is widely used because it offers good uniformity and repeatability over large areas, and the printed film thickness can be tuned through cell geometry, ink rheology, and printing speed. For TCE/TCF fabrication, gravure printing has been extensively explored for metal‐nanowire/nanoparticle lines, conductive grids, and hybrid transparent films on flexible substrates, enabling practical control over transparency–conductivity trade‐offs via printed linewidth, pitch, and ink loading. For instance, Park et al. used the gravure printing method to fabricate patterned AgNW conducting lines on a transparent flexible substrate for use in optical devices [151]. First, AgNW ink was tuned for the study, followed by doctor blading it into the grooves of a copper plate. This was followed by using a silicone roller to transfer the ink to the substrate, forming well‐defined conductive Ag lines. With all the optimized parameters, an optical transmittance of 95% was obtained for an electrode gap of 100 µm and a line resistance of 32 Ω/mm when dried at 90°C. Huang et al. used gravure printing for the fabrication of AgNW‐based large‐scale printed electronics [152]. By tailoring the properties of the water‐based AgNW ink and optimizing other printing parameters, they were able to print large‐area AgNW patterns with a fine resolution of 50 µm at a conductivity as high as 5.34 × 10^4^ S/cm on a PET substrate. The fabricated TCE was stable under multiple bending cycles and had a good optical transmittance. In another study, Seetharamiahsrinivasaraju et al. explored the use of diamond‐like carbon (DLC) engineered flat gravure surface for replacing chrome for the fabrication of conductive line electrodes on flexible substrates [153]. The study concluded that electrode grids using standard DLC increased the line widths for 20 and 30 µm lines compared to chrome. Additionally, the TCEs on a clear PET substrate had a lower line resistance than chrome, even when using different conductive inks, and had optical transmittance of >90% for line space > 500 µm and >70% for line space > 200 µm. Gravure printing has also been employed for the scalable fabrication of electrochromic devices, like the roll‐to‐roll flexo‐graphical printing of PEDOT:PSS electrodes for a paper‐based self‐powered electrochromic device [111]. An ink based on IPA, PEDOT:PSS, and DMSO was optimized and then gravure printed on a paper substrate, followed by electrolyte coating and a Zn electrode to fabricate an electrochromic device. The device had good coloration contrast, achieving a maximum optical contrast of 0.27 at 650 nm, confirming its strong visible‐light modulation performance.

Gravure‐offset printing is a closely related variant of gravure printing that improves print fidelity by using an intermediate compliant blanket (offset roll) to transfer the ink from the engraved cylinder to the substrate. In this approach, ink is first picked up from the gravure cylinder (after doctor‐blading) onto the blanket and then offset‐transferred to the target substrate, reducing defects and allowing reliable printing on rough, soft, or flexible surfaces. Gravure‐offset is widely adopted because it enables finer features and smoother printed patterns with improved edge clarity compared with direct gravure, while also being compatible with high‐speed manufacturing. It has been frequently used to fabricate “invisible” metal grids/meshes and polymer–metal hybrid TCEs/TCFs, where accurate control of grid geometry (line width and spacing) is critical for maintaining high transmittance, low haze, and mechanical durability. In our laboratory, gravure‐offset printing was used by Jin et al. to fabricate Ag paste grid lines over a PEDOT:PSS layer, followed by NOA63 coating to decrease the sheet resistance of the TCE serving as a back electrode for flexible strip OSC [120]. The inclusion of the embedded Ag grid lines brought down the sheet resistance from 107 to 11Ω/□ for a line gap of 90 µm. The transmittance of the electrode also increased from ∼49% for a line gap of 90 µm to 63% for 190 µm while maintaining similar sheet resistance and was also mechanically stable for 5000 bending cycles. In another of our laboratory's works, Ko et al. fabricated the back electrode of a strip‐shaped OLED by embedding an Ag electrode in a PEDOT:PSS layer as a hybrid fiber TCE using the gravure‐offset printing technique [29]. The TCE fabricated using this technique had a low sheet resistance of 0.9 Ω/□ at 100 µm of Ag line pitch with a transmittance of 45%, which increased to 65% for 200 µm of Ag line pitch (sheet resistance 2.4 Ω/□). Ohsawa et al. fabricated an “invisible” Ag‐grid/PEDOT:PSS TCE on flexible epoxy film through gravure‐offset printing of the Ag grid for use in an electroluminescent device [109]. With an electrode width of ∼5 µm, spacing of 200 µm, and a grid thickness of 0.6 µm, this electrode had a transmittance of ∼80% using epoxy film as compared to a lower for PEN film. The TCE had great mechanical flexibility, as the resistance change was found to be minimal for over 20 000 cycles. Finally, the TCE was successfully used to fabricate a commercial alternating‐current powder electroluminescent device. Kim et al. reported a reverse gravure‐printing technique for micropatterning of CuNW for the fabrication of TCEs [154]. In the work, first, a semi‐dried ink film of CuNWs was coated over a silicone blanket through Mayer coating, followed by transferring onto a patterned intaglio trench by rolling a hand roller over it. Finally, in order to create the Cu micropatterns, the CuNW grids were finally moved from the hand roller onto the target substrate and exposed to a laser. The obtained TCE achieved a sheet resistance of 31.6 Ω/□ and a high optical transparency of 79% at 550 nm, and was further applied to flexible LEDs and transparent heaters. Overall, gravure and gravure‐offset printing are scalable roll‐to‐roll routes for producing large‐area TCE/TCF patterns with repeatable control over film thickness and grid geometry.

Gravure printing is one of the best routes for roll‐to‐roll TCE production with high‐throughput because of its high printing speed, repeatable film thickness, and ability for high‐area coating. The only disadvantage is the requirement for the pattern to be engraved onto the cylinder, which needs high initial tooling costs and thus reduces design flexibility. Apart from this, the ink transfer also depends on the cell filling and doctor blading, leading to pinholes and variations in line dimensions. Hence, gravure is most suitable for established device structures and less suitable for prototyping. Future development should focus on finer engraving, better nanoparticle transfer and lower temperature curing, and defect monitoring to produce finer and smoother conductive grids.

### 3.4 Screen Printed TCEs

Screen printing is one of the facile printing technologies characterized by its lower cost, scalability in production, and higher efficiency in operation. It involves forcing a viscous ink through a patterned mesh stencil by a squeegee onto a substrate, followed by annealing to form the desired pattern (Figure 5d). It is a widely used technique because of its excellent pattern control and easy operation. It has been overly used for the fabrication of grid and hybrid film‐based TCEs and TCFs, including flexible ones, as it is excellent at controlling line width, spacing, and ink loading. Li et al. introduced a series of water washing steps to improve the conductivity of screen‐printed AgNWs TCFs [155]. Finally, the AgNWs TCF with a low sheet resistance of 0.72 Ω/□ was obtained through a combination of plasma treatment for 15 min at 180 W power, a high annealing temperature of 110°C and high mechanical pressure of 35 MPa. Wagner et al. increased the mechanical robustness of TCE while still maintaining a low sheet resistance through the inclusion of a slot die‐coated Boron Nitride Nanotube interlayer before the screen‐printed AgNW layer [156]. Following this process, the screen‐printed AgNW hexagonal microgrids had an optical transparency between 65%–89% on a PET substrate with a low sheet resistance of 2.9 Ω/□. The inclusion of Boron Nitride also improved the thermal performance of the TCE, leading to highly stable TCEs for optoelectronic applications. Lin et al. efficiently combined screen printing and vacuum filtration for the patterning of an AgNW network for fabricating a TCE [157]. In the study, screen printing was used to develop a dyed PDMS mask over the filtration membrane in the form of a pattern negative, followed by the filtration of AgNWs through it to fabricate the final TCE. Even at a low AgNW density of 12.5 µg/cm^2^, the TCE achieved a low sheet resistance of 7.3 Ω/□ while still possessing an optical transmittance of 79.6% (at 550 nm). Overall, screen printing is a practical and robust route for fabricating patterned TCEs and TCFs; fast, affordable, and easy to scale to large areas. With the right ink and post‐treatment, it can deliver reliable conductivity while keeping films reasonably clear.

Screen printing is suitable for thick and highly conductive metal grids as it combines high throughput, inexpensive equipment, large‐area compatibility, and high‐viscosity inks. However, the resolution is mainly controlled by mesh dimensions, ink spreading, ink thickness, and substrate‐screen separation. The requirement of high‐solid inks leads to thick and rough inks, which makes the process less compatible with OLEDs, photovoltaics, and other devices containing ultrathin functional layers. Repeated printing can also cause screen wear and gradual changes in feature dimensions. Hence, this is attractive for conductive and simplistic manufacturing rather than sub‐micrometer resolution and smooth surfaces. Finer meshes, ink‐rheology engineering, and improved control for edge spreading are some routes toward higher‐resolution transparent electrodes.

### 3.5 3D Printed TCEs

3D printing has become a go‐to technology for fabricating highly conductive, scalable TCEs and TCFs in recent years [158]. 3D printing (Figure 5e) or Direct Ink Writing (DIW) is one of the widely used 3D printing techniques for the fabrication of flexible electronics. For instance, Zheng et al. fabricated graphene grid patterns using an optimized ink formulation containing sodium dodecyl sulfate (SDS) and ethanol alongside graphene [159]. The as‐fabricated grid pattern achieved a sheet resistance of 21.3 Ω/□ and light transmittance of 68.5%, while demonstrating good thermal and corrosion resistance. The SDS omits the coffee‐ring effect due to effective Marangoni flow, whereas ethanol promotes a well‐ordered arrangement of graphene flakes during hot pressing at 300 mPa for 5 min after printing. Kong et al. used DIW to fabricate an Ag nanowire grid with thin Ag lines of 33 µm while still attaining a sheet resistance of 25.3 Ω/□ at a visible‐light transmittance of 98.6% [160]. In the study, the group used a capillary tube of inner diameter of ∼30 µm as the printing nozzle, which allowed them to fabricate a large area of 220 × 160 mm^2^ orthogonal grids with a relatively low area density of AgNWs (∼2.1 µg/cm^2^). Finally, it was used to design a flexible, transparent heater having good Joule heating performance.

DIW provides excellent control over electrode geometry and helps in attaining three‐dimensional and high aspect ratio metal grids without proportionally increasing optical shadowing. High‐resolution printing through DIW often requires the control of ink rheology, printing nozzle, printing pressure, and drying kinetics and thus often results in nozzle clogging, filament spreading, discontinuous extrusion, layer misalignment, and surface roughness leading to electrical non‐uniformity. Hence, DIW is more suitable for customized grids, three‐dimensional architectures, and multifunctional devices rather than conventional high‐volume planar coating. For commercialization smaller‐nozzle printing, multi‐material system, low‐temperature sintering, and ink with shear‐thinning followed by good shape recovery are needed for DIW.

### 3.6 Blade Coating of TCEs

Apart from DIW, other coating technologies, such as D‐Bar coating, Blade coating, Mayer rod coating, etc., are also common strategies recently employed for the fabrication of TCEs and TCFs. A TCE can be produced using a blade‐coating technique, in which a liquid containing conductive materials is evenly spread across a substrate. The blade determines how thick or thin the resulting film will be. The network of conductive particles provides electrical pathways, while the film's thinness allows light to pass through, making it transparent (Figure 5f). Huang et al. used blade coating to sequentially deposit AgNW and a transparent polymer over PET substrates to obtain large‐area, ultra‐flexible TCEs [161]. This TCE showed a transparency of 78% at a low sheet resistance of 10 Ω/□ and showed almost no conductivity loss over 10000 bending cycles, while also maintaining the same over 60 days. In another innovative study, Zhu et al. used a blade‐coated polystyrene (PS) suspension in water as a precursor for fabricating an Au micromesh‐based TCE [162]. In the study, after blade‐coating and annealing the PS layer on the PET substrate, they deposited a sacrificial Al layer by thermal deposition. Subsequently, the PS layer was dissolved in toluene, and an Au layer was deposited by thermal evaporation. This was followed by washing with 10% HCl to remove the Al islands, resulting in Au interconnected network that duplicates the first polymer networks, which had a transmittance of 86% and a conductance of 80 Ω/□, and was later applied to fabricate OLEDs. In their study, Wei et al. used a series of micrometer‐sized razor blades to plow parallel microgrooves into a PC film, followed by blade coating and filling the grooves with Ag paste [163]. This was followed by sandwiching between two PVB films and hot pressing, resulting in a TCE with a transmittance of 78.2%, a sheet resistance of 3.1 Ω/□, and high bending stability (depicted by sheet resistance variation over 1k bending cycles. Interestingly, Guo et al. developed blade‐coated MXene TCFs through the rational design of MXene inks having high conductivity of 19325 S/cm while maintaining transmittance of 83.4% (≈6.7 nm) [164]. They used a combination of large flakes of ∼12.2 µm and normal flakes of ∼1 µm lateral sizes to design MXene inks and then used blade‐induced shear force followed by heat treatment to develop TCFs for application in Joule heaters and symmetric micro‐supercapacitors. Gholami et al. emphasized that the electroplating of Au over AgNW TCE prepared by spray‐coating AgNW and blade‐coating PS over it not only decreased the sheet resistance but also increased its chemical stability [165]. Blade coating has also been used to passivate TCEs for enhancing PCE in PSCs. For instance, Liu et al. studied the effects of blade‐coated mono‐ethanolamine passivation over the blade‐coated AgNW/SnO_2_ composite TCE and found that the PCE increased from 12% to 13.5% because of the reduction of the surface hydroxyl groups [107].

Blade coating is a reliable method for a simple, material‐efficient, and large‐area‐compatible technique for coating of continuous CNT, graphene, MXene, PEDOT:PSS, and nanowire films. However, has limited patterning capability and requires the aid of masks or additional patterning steps for printing patterns. Film uniformity can be controlled by controlling blade gap, ink viscosity, coating velocity, drying conditions, and substrate wetting, as well as edge defects and thickness gradients. Future work should thus focus on meniscus control, shear‐induced alignment of 1D and 2D nanomaterials, slot/blade‐compatible rheology, rapid drying without aggregation, and integration with continuous roll‐to‐roll patterning.

### 3.7 Spray Coating of TCEs

Spray‐coating is an excellent choice for depositing an organic/inorganic material because it is inexpensive and compatible with existing roll‐to‐roll (R2R) assembly‐line production (Figure 5g). Spray coating is another widely used technique for fabricating TCEs for various applications [102, 166]. Poobalan et al. used spray‐coating to deposit high‐quality AgNWs and develop TCE for use in PSCs, with their optimized AgNW‐based TCE having a sheet resistance of 18.1 Ω/□ with enhanced optical properties [167]. Similarly, Chen et al. developed a spray‐coating for the development of large‐area (15 × 15 cm) stretchable TCEs with a surface resistance of 70 Ω/□ at a transmittance of 85% based on DMSO‐modified PEDOT: PSS ink [168]. Studies have also shown that the properties of the TCE can be enhanced by further modifying the spray‐coated initial layer. For instance, Khan et al. fabricated a SnO_2_ coating by Atmospheric‐Pressure Spatial Atomic Layer Deposition (AP‐SALD) on spray‐coated AgNW on a glass substrate to create TCEs for the deposition of V_2_O_5_ thin films for electrochromic applications [169]. Wu et al. showed that ethyl cellulose can act as an auxiliary agent in the spray coating of AgNWs on a curved surface to prepare flexible TCEs with an average sheet resistance of 8.9 Ω/□ at a high transparency of 92.33%, and good flexibility [170]. Interestingly, Sun et al. used the coffee ring effect of the spray‐coated AgNWs on PDMS to create AgNW bundle micro‐meshes embedded in polyimide TCFs with high flexibility, corrosion stability, and high thermal stability [171]. Fox et al. used the superhydrophilicity of AgNWs dispersion to form coffee‐ring‐effect‐free stable TCFs through further spray coating of a GO layer and deposition of Au [172]. The sample with a resistivity of 250 Ω/□ exhibited a transmittance of 97.7%, whereas the sample with a resistivity of 24.7 Ω/□ had a transmittance exceeding that of immaculate glass. Apart from AgNWs, Navik et al. synthesized a graphene nanosheet‐CuNWs composite for the fabrication of spray coating‐based TCE over a PET substrate, which also had excellent oxidation stability at 65°C for 46 days [173]. The TCE thus fabricated exhibited 87% transmittance at a resistance of 81.42 Ω/□ and demonstrated extended flexibility over 1000 cycles, suggesting the potential for other metal‐based TCEs. Alternatively, Kim et al. used ultrasonic spray pyrolysis to fabricate antimony‐doped tin oxide‐based films for use as TCEs [174]. By adding the dopant to the spray solution on a heated substrate (>400°C), they achieved a TCE with transmittance values in the visible spectrum of 80%–90% and sheet resistance of 10–20 Ω/□, indicating the effectiveness of the process. Another aspect of fabricating TCFs is achieving super‐hydrophobicity alongside high transparency and conductivity. For this, Torun et al. first grafted a layer of polyethylene glycol onto ITO glass and then spray‐coated a layer of alkyl silane‐functionalized nanoparticles [175]. The TCFs thus obtained had a high‐water contact angle, transparency of 94% at a sheet resistance of 13.05 Ω/□, and high stability.

Although spray coating has many advantages, such as low equipment cost, large‐area covering, and broader ink compatibility but is limited due to high material utilization. Further, the droplet size distribution, nozzle distance, substrate temperature, and solvent evaporation influence film formation. Also, the pattern resolution is also comparatively poor unless masks are employed. Thus, this is suitable for large‐area TCEs and not high‐resolution patterns, and needs control of electrostatic spray control, ultrasonic atomization, and in‐line optical/electrical feedback for improving the reproducibility and material utilization.

### 3.8 Bar Coating of TCEs

For roll‐to‐roll production of TCEs, bar coating (such as Mayer‐rod/wire‐wound rod) is an easy, affordable, and scalable way to deposit uniform nanomaterial networks (AgNWs, CNTs, and graphene hybrids) over large areas. Additionally, it allows for practical tuning of the conductivity–transparency trade‐off by providing good control over film thickness, coverage, and haze through rod size and ink rheology (Figure 5h). Noh et al. used a 2‐step bar‐coating process to fabricate a grid‐like AgNW‐based large‐area stretchable transparent electrode (STCE) with a sheet resistance of ∼25 Ω/□ and having ∼85% transmittance at 550 nm [176]. In their process, they first bar‐coated the first AgNW film on a glass substrate with AgNWs aligned in the direction of coating, followed by a second layer orthogonal to it. Finally, they added TPU over the film and lifted off to get the STCE. Mi et al. proposed modifying AgNW ink by incorporating hyperbranched molecules as both dispersant and stabilizer [177]. This resulted in thixotropic AgNW‐based inks, containing a solid loading of 20%, when coated by bar coating, yielding a TCE with a sheet resistance of 17.1 Ω/□ at a transmittance of 94.7% transmittance, demonstrating the significance of the hyperbranched molecules. In our lab, Ovhal et al. used the D‐bar coating process to fabricate modified PEDOT:PSS‐based TCEs with a conductivity of 3645 S/cm at an optical transmittance of 85%, comparable to spin coating [75]. The D‐bar coating process comparison with spin coating for the fabrication of uniform TCEs. The thickness variation with *R_sheet_*, and the bar‐coated TCEs achieved a lower sheet resistance than spin coating. The process was then used to fabricate a large‐area (64 cm^2^) semi‐transparent supercapacitor for transparent and flexible energy‐storage devices. Lahane et al. showed that a Mayer rod coating of an AgNW layer on ITO/ZnO/P3HT photodetector layers as the top electrode yields a higher‐transparency layer with a similar sheet resistance to the spin‐ and spray‐coating methods [178]. The Mayer rod‐coated AgNW had a sheet resistance of 24 Ω/□ and a light transmission of 91%, and also showed a higher photosensitivity of 1500 (for the ITO side) and 630 (for the AgNW side) at 525 nm.

Bar or Mayer‐rod coating is attractive for low‐cost, high‐speed deposition of uniform conductive networks and is highly compatible with continuous roll‐to‐roll manufacturing. However, achieving thickness and morphology are strongly dependent on rod geometry, ink viscosity, wetting, coating velocity, and solvent evaporation. Nanowires or high aspect ratio fillers may align or aggregate during coating, leading to anisotropic conductivity. Surface defects in the substrate or coating bar propagate over large areas. As like blade coating, bar coating has limited patterning capability. Future work should focus on rheological formulations suppressing aggregation, controlled alignment where anisotropy is an advantage, multi‐pass deposition with minimal optical loss, and integration with patterned or selective‐coating techniques.

### 3.9 Layer‐By‐layer Printing of TCEs

Layer‐by‐layer (LBL) or multilayer printing of TCEs is a process where multiple thin layers of conductive materials are deposited one after another onto a substrate (Figure 5i). By carefully stacking these layers, the electrical, optical, and mechanical properties of the electrode can be precisely tuned to achieve the desired performance. DIW has been employed in our lab to increase the performance of semi‐transparent perovskite solar cells (ST‐PSCs) using sputtered ITO and multilayered Ag mesh as top 3D TCEs, as shown in Figure 6a [65]. Tyagi et al. fabricated a TCE of Ag mesh sub‐electrodes having an optimized aspect ratio over sputtered ITO (Figure 6b), achieving an < 1 Ω/□ even at a transparency of ∼85% as a top electrode (Figure 6c), delivering a high PCE of 16.26% for a single cell. In advancing high‐performance smart textile electronics, flexible TCEs without ITO are especially advantageous for their greater flexibility and lower production costs compared to brittle ITO‐based equivalents. In another work from our lab, Ovhal et al. fabricated ITO‐free DIW‐printed PEDOT:PSS/Ag TCEs (Figure 6d) for use in flexible organic solar cells [64]. As is evident from the sheet resistance variation in Figure 6f, the strategy resulted in a highly conducting TCE (with sheet resistance ∼ 4 Ω/□) and transmittance of >80%. Finally, they used it to assemble a free‐standing, one‐dimensional stripe‐OSC (a digital image of the device is presented in Figure 6e) that achieved a maximum PCE of ∼9.3% for a textile‐based application. In another innovative study, Park et al. combined 3D printing and electrospinning to fabricate 3D‐stacked (Figure 6g), self‐aligned nanofibers with up to 80 layers [179]. Figure 6h shows the FESEM images of the different printed stacked nano‐walls. As shown in Figure 6i for the resistance vs number of the 3D‐printed layers, the resistance decreased to < 40 Ω for over 80 layers. They modified the conductivity of the ink before printing and, using transparent and flexible polydimethylsiloxane films embedded with Ag‐sputtering, fabricated a TCE with a very high transmittance of 98.5% and average resistivity of 3 × 10^−7^ Ω m, comparable to 2D‐printed Ag ink (2 × 10^−7^ Ω m).

**FIGURE 6:**
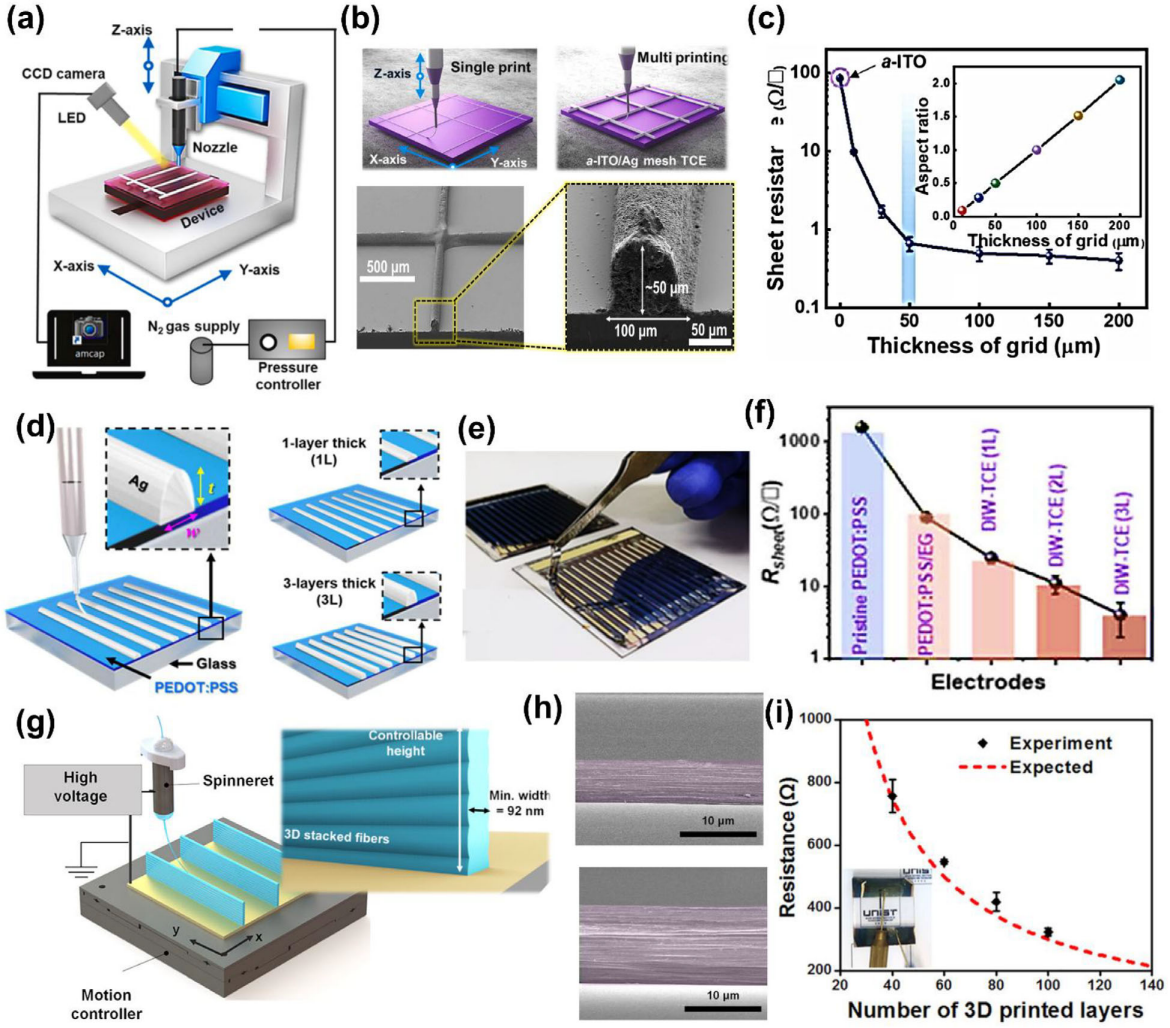
(a) Schematic illustration of the DIW instrument and (b) the multilayer printing of Ag mesh along with its FESEM image. (c) Plot showing the variation of sheet resistance with the thickness of Ag mesh for the TCE. Reproduced with permission [65]. Copyright 2022, Elsevier. (d) Schematic illustration of the layer‐by‐layer Ag conducting lines over the PEDOT layer. (e) The digital image of the stripe‐shaped OSCs. (f) The *R_sheet_* variation of the reference and printed TCEs. Reproduced with permission [64]. Copyright 2023, Elsevier. (g) Schematic and (h) SEM images of 3D‐printed nano‐walls showing the number of layers. (i) Plot showing variation of *R_sheet_* with the number of 3D printed layers. Reproduced with permission [179]. Copyright 2020, American Chemical Society.

The brittleness of ITO thin films often poses a challenge for the fabrication of flexible TCFs. Hence, studies have mainly focused on the fabrication of AgNW‐based thin films, with the layer‐by‐layer approach among the key contenders. Qian et al. utilized an all‐solution, layer‐by‐layer deposition of 3,4‐dimethylbenzoic acid (PA) and tannic acid (TA) (PATA)‐ modified PEDOT:PSS, with an AgNW film in between, via spin coating for the fabrication of the TCF [180]. PA‐ and TA‐doped PEDOT:PSS, enhanced the conductivity of PEDOT:PSS, while also introducing a high amount of phenol hydroxyl groups, forming hydrogen bonds, leading to AgNWs being firmly anchored in the middle. With a surface resistance of 3.1 Ω/□, the film demonstrated its maximal photoelectric value at 70% light transmission. Yang et al. studied the effect of ionic liquid on the fabrication of MXene‐AgNW‐based TCFs through the creation of ordered layered structures [181]. This method places a strong emphasis on the use of hydrophobic, nonvolatile ionic liquids that create stable surfaces by lowering interfacial energy and avoiding nanomaterial sedimentation loss, resulting in superior optoelectronic qualities (9.4 Ω/□ sheet resistance and 93% transmittance). Shin et al. used the LDL method to prepare an MXene, AgNW, and colorless polyimide‐based TCE with a 79% transmittance at 550 nm and a considerably lower sheet resistance (13.08 Ω/□) for use as a flexible TCE in solar cell fabrication [182]. Oh et al. used the LBL method to fabricate a TCE with a Ru metal and RuO_2_ subsurface layer via thermal reduction after coating [183]. The thin film of ∼6nm exhibited a high transmittance of 79% in the visible range and demonstrated a high figure‐of‐merit at approximately 3.4 × 10^−4^ Ω^−1^. Kwon et al. showed that sequential LBL coating of bio‐renewable chitin and cellulose nanofibers can be used to increase the durability of the AgNW‐based TCE against harsh conditions like 40 min treatment of UV/O_3_, thermal treatment at 250°C for 350 min, and other chemical resistance, while maintaining good flexibility, conductance, and transmittance [184].

The principal limitation of this technique is increased manufacturing complexity, i.e., every additional deposition requires drying, treatment, or alignment step, which increases processing time and can create another interface at which contact resistance, delamination, solvent incompatibility, or chemical degradation may occur. Multilayer structures may therefore offer outstanding performance at the laboratory scale but may not be economically scalable for high‐throughput manufacturing. Future approaches should minimize the number of required layers, use orthogonal solvent systems and compatible thermal budgets, and design individual layers to have multiple functions simultaneously.

### 3.10 Other Methods

In addition to the methods mentioned above, several others have been used to fabricate high‐transparency, high‐conductivity TCEs and TCFs. Duan et al. used a maskless direct‐write photolithography process to deposit Au grid lines on glass for OSC applications [185]. The study showed that a pitch of 400 µm Au grid had a sheet resistance of 74 Ω/sq at an average visible light transmittance of 95.7%, better than an 80 nm ITO film at 71 Ω/sq at an average visible light transmittance of 84.7%. In recent years, research has focused on fabricating uniform single‐layer MXene films with continuous, dense conductive pathways for various applications. In this regard, Xue et al. employed the Langmuir–Blodgett (LB) technique to fabricate a single‐layer Ti_3_C_2_T*_x_* MXene film with MXene flakes of ∼52 µm^2^ over a variety of substrates (glass, PET, etc.) [186]. The LB film was formed by adding methanol to the MXene dispersion, then spreading it at the air‐water interface. This process enabled them to fabricate MXene films with controlled morphologies, with a sheet resistance of 318 Ω/□ and a transmittance of 88%, for applications in various next‐gen electronic devices. The transfer of CVD‐grown graphene and CNT films onto a flexible, transparent substrate in a wrinkle‐free manner has been a significant challenge for the fabrication of TCEs and TCFs. To solve this issue, Zhang et al. developed a wet transfer printing process in which they use a glass frame on top of the etchant solution, forming a convex surface [187]. Then the Cu was etched on this convex surface and, with the aid of a liquid protection layer of n‐hexane, transferred to the target substrate. Through this process, they prepared graphene heterostructures on different substrates, and a 4‐layer graphene‐based film achieved a low sheet resistance of 200 Ω/□ at near 100% transmittance, reiterating the importance of the process for making graphene film‐based TCEs. Ishizaki et al. used a film‐transfer technology to fabricate SWCNT thin films acting as TCEs via a wet transfer protocol [188]. In the study, an all‐solution process strategy in which filtered SWCNTs were transferred onto a PET substrate through dipping in suitable solvents was used to prepare SWCNT‐based TCEs. The transferred films show a low sheet resistance of ∼400 Ω/□ at 90% transmittance, which decreases to ∼50 Ω/□ at 65%, and they have further applications in PSCs. In another interesting study, Li et al. fabricated a Ti_3_C_2_T_x_‐based TCE using the film‐transfer technique: they prepared a dispersion of MXene sheets in IPA, formed a thin layer of MXene sheets at the n‐hexane‐water interface, and then transferred it onto a PET substrate [189]. The TCE with a self‐assembled MXene film exhibited sheet resistances of 1623, 210, and 123 Ω/□ at transmittances of 96.9%, 94.1%, and 87.8%, respectively. Further, they used the same technique to coat a TiO_2_ film over the MXene film for electrochromic application. In addition to these methods, another popular process is solution shear, which involves dropping the material in solution form between two movable film‐coating scrapers on the substrate while heating occurs below. Surface tension causes a meniscus‐like curved gas‐liquid interface to form, and when the heater forms the film, the meniscus slides over the substrate. This is how the process operates. Wang et al. used this method to fabricate a PEDOT: PSS‐based TCF with three‐layer films as the optimized condition, having a thin‐layer resistance of 34.7 Ω/□, a transmittance of 93.2%, and an FoM of 151.9, and later used it to fabricate a flexible OLED [190]. Hu et al. used the strong shear force generated by the high‐speed rotating dispersion to deposit aligned AgNWs on a transparent substrate [191]. They found that at a shear rate of 4000 rpm, two orthogonally aligned layers of AgNWs can be developed, leading to stable electrical performance up to 12 V rather than 8 V for randomly arranged AgNW TCF. Overall, these new fabrication routes demonstrate that high‐performance TCEs/TCFs can now be engineered by adjusting ink rheology, nanoscale alignment, and interfacial adhesion, rather than relying solely on brittle ITO. They range from scalable coating methods (blade/bar/spray) to assembly/transfer and shear‐based approaches.

The commercialization of printing technology depends on trade‐offs among resolution, throughput, ink compatibility, pattern flexibility, material utilization, and required film thickness. Inkjet printing provides maskless control and low waste but has a narrow ink‐property window and is also susceptible to nozzle‐ and drying‐related defects. EHD offers high direct‐ink‐writing resolution but adds complexity and reduces throughput. Gravure printing is roll‐to‐roll processibility and reproducible but needs detailed patterning tools, whereas screen printing can work with highly conductive, viscous inks at high throughput but generally results in thicker and lower‐resolution features. DIW has good control over 3D‐architecture and aspect ratio but remains relatively slow because of its one‐by‐one serial printing. Blade, bar, and spray coating are attractive for large‐area continuous films, although their direct patterning capability is limited. Layer‐by‐layer processing shows the greatest compositional and interfacial flexibility but suffers from an increased number of fabrication steps and potential failure interfaces. Therefore, the most suitable process for the fabrication of a TCE is a combination of the ink rheology, desired architecture, throughput, substrate compatibility, post‐treatment processes, and specific surface roughness requirements. Table 1 provides comprehensive process selection criteria for each printing technique.

**TABLE 1:** Process selection criteria for printing‐based TCE.

Printing method	Typical ink requirement	Resolution	Throughput/scalability	Suitable TCE materials	Main advantages/suitable applications
Inkjet printing	Low viscosity, controlled surface tension, small particles	High	Moderate; digitally scalable	PEDOT:PSS, AgNPs, MXenes, graphene	Maskless patterning; OLEDs, PeLEDs, PVs, sensors
EHD printing	Low–moderate viscosity, electrically stable ink	Very high	Low–moderate	Metals, nanowires, polymers, carbon materials	Ultrafine electrodes and high‐resolution metal grids
Gravure printing	Low–moderate viscosity, good wetting and transfer	Medium–high	Very high; R2R compatible	Ag/AgNW, Cu, PEDOT:PSS, hybrids	Large‐area PVs, OLEDs and flexible electronics
Screen printing	High viscosity, high solid content	Medium–low	High	Ag, AgNW, carbon inks, metal grids	Thick, highly conductive electrodes; heaters and current collectors
DIW/3D printing	High viscosity, shear‐thinning ink	Medium–high	Low–moderate	AgNW, Ag, graphene, conductive composites	High‐aspect‐ratio and 3D electrodes; wearable/energy devices
Blade coating	Low–moderate viscosity, stable dispersion	Low	High	AgNW, CNT, graphene, MXene, PEDOT:PSS	Uniform large‐area films; PVs and transparent heaters
Spray coating	Low viscosity, atomizable ink	Low–medium	High	AgNW, CNT, graphene, PEDOT:PSS, oxides	Large/irregular‐area coating; PVs and flexible electrodes
Bar coating	Low–moderate viscosity, good leveling	Low	High; R2R compatible	AgNW, PEDOT:PSS, CNT, graphene	Uniform large‐area films; wearables, sensors, energy devices

**Footnote**: TCE‐ transparent conducting electrode; PV‐ photovoltaic; AgNW‐silver nanowire; CNT‐carbon nanotube; DIW‐direct ink writing; EHD‐electrohydrodynamic; R2R‐Roll‐to‐roll manufacturing.

## 4 Electronic Applications of Printed TCEs

Several studies are reviewed based on TCE developed using physical techniques such as PVD, CVD, MS, ALD, RPD, and PLD, and their applications in emerging electronics [192, 193]. In this section, we have narrowly focused on printed TCEs and their applications in emerging electronic devices. Mesh‐type or hybrid transparent conductive electrodes (TCEs) significantly improve the performance of next‐generation electronic devices, largely due to their superior conductivity. This approach encourages ongoing innovation in the development of printed TCE‐based emerging electronics for wearable and portable technologies. Key examples of advanced electronics utilizing TCEs are reviewed in this discussion.

### 4.1 Photovoltaics Applications (Perovskite, OPV, and Others) for Printed TCEs

Flexible and transparent photovoltaics stand out for their see‐through nature, lightweight construction, and robustness. To make it a real‐life use, the researchers are actively investigating innovative semiconductor materials, such as small organic molecules functioning as electron donors or acceptors, donor–acceptor conjugated polymers, and organometal halide perovskites. In addition, efforts to develop electrodes focus on TCEs, thin metal layers, or metallic nanowires, various forms of nanocarbon, and electrically conductive polymers [192, 194].

#### 4.1.1 Perovskite Solar Cells (PSCs)

Perovskites have arisen as the foremost material for semi‐transparent solar cells (ST‐SCs) due to their high PCEs, economic viability, ease of solution‐based processing, mechanical flexibility, and adjustable transparency. Research efforts continue to refine TCEs and device architectures to further enhance both the efficiency and optical properties of these devices. Ultimately, the choice of top electrode materials remains a pivotal factor impacting the overall performance and transparency of ST‐PSCs [195]. However, very few works are available on TCE‐based PSCs, motivated by the emerging device design strategy, which could be a better option for building integrated photovoltaics (BIPVs). Barkha et al. applied a scalable approach to fabricating ST‐PSC (Figure 7a) by utilizing an Ag‐metal‐based TCE composed of a sputtered amorphous (α)‐ITO film and Ag mesh sub‐electrodes produced through a 3D printing technique [65]. With an optimized AR of 0.5, the 3‐dimensional TCE achieves a *R_sheet_* below 1 Ω/□ and a T(%) of approximately 85%. When used as the top contact, both standard (0.07 cm^2^) and large‐area (1.0 cm^2^) ST‐PSCs demonstrate PCE of 16.26% and 15.52%, respectively, with over 85% (at NIR range), as illustrated in Figure 7b,c. Furthermore, these ST‐PSCs exhibit improved stability (ambient & photostability) compared to evaporated Ag‐based perovskite solar cells, owing to the α‐ITO buffer layer, which inhibits moisture ingress and ion migration (Figure 7d). When implemented as top cells, the standard 4‐terminal ST‐PSC/Si‐SC tandem cells achieve PCEs of 26.47%. In the case of ST‐PSCs, the fabrication method remained consistent, except that α‐ITO or α‐ITO/Ag mesh was used instead of an Ag film for the top contact. To assess the effectiveness of the α‐ITO/Ag 3D TCE mesh, p‐i‐n‐type opaque PSCs were fabricated using a single‐step antisolvent process. The highest‐performing opaque PSC (area ∼0.07 cm^2^) reached a PCE of 17.20%, which improved to 17.77% upon applying an ARC. Opaque PSCs here performed among the best for wide‐bandgap perovskite films. ST‐PSCs with α‐ITO had a lower PCE (12.73%) due to high sheet resistance, whereas those with α‐ITO/Ag mesh reached 16.04% due to improved JSC and FF. The small PCE drop (∼1.5%) for ST‐PSCs compared to opaque devices confirms the α‐ITO/Ag mesh's effectiveness as a back contact. The scalability of the α‐ITO/Ag mesh‐printed ST‐PSC was examined by fabricating larger‐area devices (Figure 7e). The Ag mesh was constructed by sequentially printing five layers, ensuring accurate placement and rapid drying of each layer to maintain structural integrity (Figure 7f). This method enhanced the mesh's thickness while keeping its width unchanged, which resulted in a higher aspect ratio for the 3D TCE α‐ITO/Ag mesh. ST‐PSCs with an α‐ITO/Ag mesh maintained a PCE of 12.83% for large areas up to 2.7 cm^2^, retaining approximately 79% of their efficiency as device size increased (Figure 7g). In comparison, large‐area devices utilizing only α‐ITO experienced a PCE decline below 1%. In opaque PSCs, aging led to Ag diffusion into the active layers, but the α‐ITO barrier layer prevented this issue in ST‐PSCs. The presence of α‐ITO also suppressed Br migration, thereby enhancing the long‐term stability of ST‐PSC devices. The PCE of tandem cells was evaluated using a 2.5 × 2.5 cm^2^ optical filter designed to replicate the ST‐PSC layers (inset of Figure 7h). Individually, Si‐SCs and ST‐PSCs demonstrated PCEs of 20.07% and 16.26%, respectively, while the Si‐SC under filtered conditions decreased to 10.21% due to reduced J_SC_ (Figure 7h). Employing an a‐ITO/Ag mesh as the top electrode facilitated the development of high‐performance and scalable ST‐PSCs and tandem devices. Figure 7i shows that the TCE could also be used in building‐ and vehicle‐integrated photovoltaics after further optimization.

**FIGURE 7:**
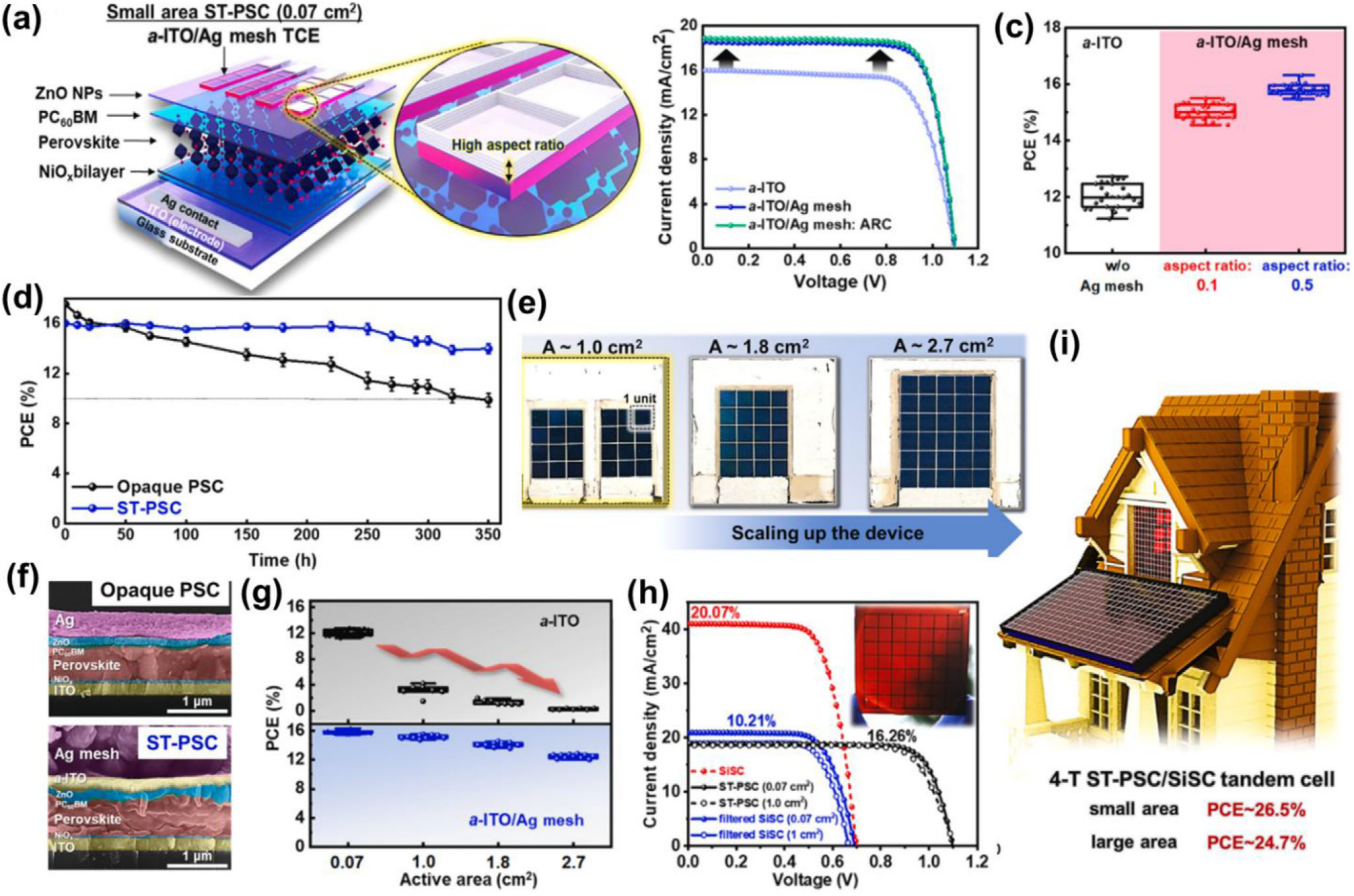
(a) Illustration showing the structure of the ST‐PSC featuring a top α‐ITO/Ag mesh contact. (b) Current–voltage (*J*–*V*) curves, (c) PCE distributions, and (d) Photostability for ST‐PSCs under 100 mW/cm^2^ white LED illumination. (e) Digital images displaying ST‐PSCs with different active areas. (f) Cross‐sectional views comparing opaque PSCs and ST‐PSC devices. (g) Box‐and‐whisker plots illustrating the reproducibility of ST‐PSCs with and without Ag mesh sub‐electrodes across different active areas. (h) Comparative *J*–*V* curves for Si‐SC, ST‐PSC, and Si‐SC with the ST‐PSC used as a filter. (Inset: Actual photograph of an ST‐PSC (with α‐ITO/Ag mesh TCE) filter employed in testing a 4‐terminal tandem solar cell.) (i) Schematic representation of possible uses for ST‐PSCs and 4‐terminal ST‐PSC/Si‐SC tandem cells in BIPVs. Reproduced with permission [65]. Copyright 2022, Elsevier,

Further, Wang et al. were among the first to fabricate PSCs utilizing transfer‐printed gold TCEs, achieving a PCE of 13.72% [196]. This performance surpassed that of comparable devices with thermally evaporated Au electrodes. Notably, after 97 days of storage under nitrogen without encapsulation, the PSCs with transfer‐printed TCEs retained 71% of their initial efficiency. Further. PSCs were fabricated on hybrid TCEs with 1‐inch by 1‐inch dimensions. These TCEs were used directly, without additional cleaning or ultraviolet ozone (UVO) treatment. To facilitate processing and handling, flexible substrates were placed on thin glass supports with a thickness of 0.5 mm. The devices were constructed using a p‐i‐n architecture. From bottom to top, the layers included hybrid TCE, neutral PEDOT:PSS, regular PEDOT:PSS, MeO‐2PACz, MAPbI_3_, PCBM, BCP, and Al/Ag as top contacts [197]. For devices incorporating the neutral/regular PEDOT:PSS bilayer and MeO‐2PACz, the process began with spin‐coating neutral PEDOT:PSS at 4000 RPM for 30 s, followed by annealing at 120°C for 10 min on the PET/TCE substrate. Next, MeO‐2PACz was spin‐coated at 3000 RPM for 30 s onto the PEDOT:PSS layer, and the resulting film was annealed in a nitrogen glovebox at 100°C for 10 min. The MAPbI_3_ layer was then deposited by spin‐coating at 5000 RPM for 20 s and annealed at 100°C for 10 min in a purge box. Afterward, the PCBM layer was spin‐coated at 1200 RPM for 60 s, and the BCP layer was applied at 4000 RPM for 30 s. Finally, the top metal contacts comprising 100 nm of aluminum and 50 nm of silver were deposited by thermal evaporation in a nitrogen atmosphere. A total of six diodes, each measuring 0.11 cm^2^ in area, were fabricated on individual substrates sized 1 inch by 1 inch. PSCs constructed using both 9‐pulse and 1‐pulse hybrid TCEs exhibited comparable performance. This result demonstrates that even a single photonic curing pulse is sufficient to produce efficient hybrid TCEs. The highest power conversion efficiencies achieved were 11.4% for the 9‐pulse TCEs and 10.7% for the 1‐pulse TCEs. A new type of rear TCE using gold micro‐meshes has been developed as an alternative to ITO in PSCs. Au meshes, when optimized, can extract up to 75% of the photocurrent compared with conventional ITO‐based devices, yielding a PCE of 8.65% [44]. The use of a hybrid mesh structure combined with a 10 nm ITO layer significantly improves the PCE of PSCs, reaching 12.1%. This configuration enables over 80% current extraction compared to standard reference devices. TCEs offer an alternative to conventional opaque metal contacts in PSCs, allowing for advanced designs such as bifacial and multi‐junction devices. Furthermore, aerosol jet‐printed silver TCEs serve as effective front electrodes. When paired with a 5–10 nm gold layer, these systems achieve high PCE in semi‐transparent PSCs (up to 16.8%). Alternatively, integrating a 5–10 nm ITO layer enhances bifacial device performance without compromising efficiency. The substrate choice, particularly PET/TCE combinations, strongly influences the morphology and performance of MAPbI_3_ films in PSCs.

Among the tested options, AgNW‐IZO hybrid TCEs deliver the highest PCE and promote the formation of bigger, higher‐quality grains of perovskite, as indicated by improved structural and optical characteristics [198, 199]. PSCs utilizing AgNW‐IZO hybrid transparent conductive electrodes demonstrate enhanced performance, reaching a PCE of 11.3% ± 0.3%. This is notably higher than the PCE values of devices made with AG12 (7.04% ± 0.36%) or Sigma (7.37% ± 0.25%) electrodes. This enhancement is attributed to the hybrid TCE's lower surface energy, which benefits perovskite film growth. In contrast, devices using AG12 and Sigma TCEs show reduced PCE and less favorable grain structures at the HTL‐MAPbI_3_ interface. Overall, researchers have explored all printed perovskite solar cells in ambient air and tested their performances [200]. Some works have explored inkjet‐printed absorber layers for efficient printed PSCs as well as modules [102, 129, 201]. However, to further explore all printed ST‐PSCs under ambient conditions, the printed TCE is the best choice for developing stable, efficient devices.

#### 4.1.2 Organic Photovoltaics (OPVs)

The development of high‐performance flexible OPV relies on TCEs that offer strong optoelectronic characteristics and mechanical flexibility. Recently, our group developed PEDOT:PSS/Ag stripes with three distinct silver stripe spacings using a gravure‐offset printing method. Pattern rolls with silver stripe intervals of 90, 140, and 190 µm were pre‐engraved in the printer system, as outlined in Figure 8a. Adding a highly conductive silver metal sub‐electrode lowers the sheet resistance of PEDOT:PSS [120]. Next, one‐dimensional self‐folded organic solar cells (1D SF‐OSCs) were fabricated by precisely slicing the F‐OSCs using a surgical scalpel, as shown in Figure 8b. Figure 8c displays a schematic of a standard single‐cell organic solar cell (SC‐OSC) device structure. After the slitting step, the SF‐OSC maintained nearly the same PCE, with its current–voltage characteristics showing minimal variation from the original F‐OSC, as depicted in Figure 8d. These results indicate that the top‐down slitting technique maintains the integrity of the device and does not negatively affect its performance. Self‐charging photocapacitor (OSC‐SC) fibers were developed by stacking one SF‐OSC and seven SF‐SCs vertically. These fibers were then assembled into a self‐charging photocapacitor module using a 2 series × 4 parallel connection, making them suitable for integration into wearable devices. All fibers in the module were encapsulated and interconnected with nickel tape and silver paste, allowing the module to be woven into garments. When exposed to sunlight, the woven module charges and stores energy within the textile circuit for later use. The module achieved a photo‐charge rate of 1.03 mV/s (with a photocurrent output of approximately 7.8 mA) under standard sunlight (Figure 8e). The efficiency of a single photocapacitor fiber was measured at 5.02%, while the module exhibited efficiencies of 4.94%, 3.56%, and 3.37% at discharge currents of 3.0, 3.8, and 5.0 mA, respectively. The photocapacitor fibers and modules also showed strong mechanical flexibility and durability, supporting their practical application in future textile‐based smart electronics (Figure 8i).

**FIGURE 8:**
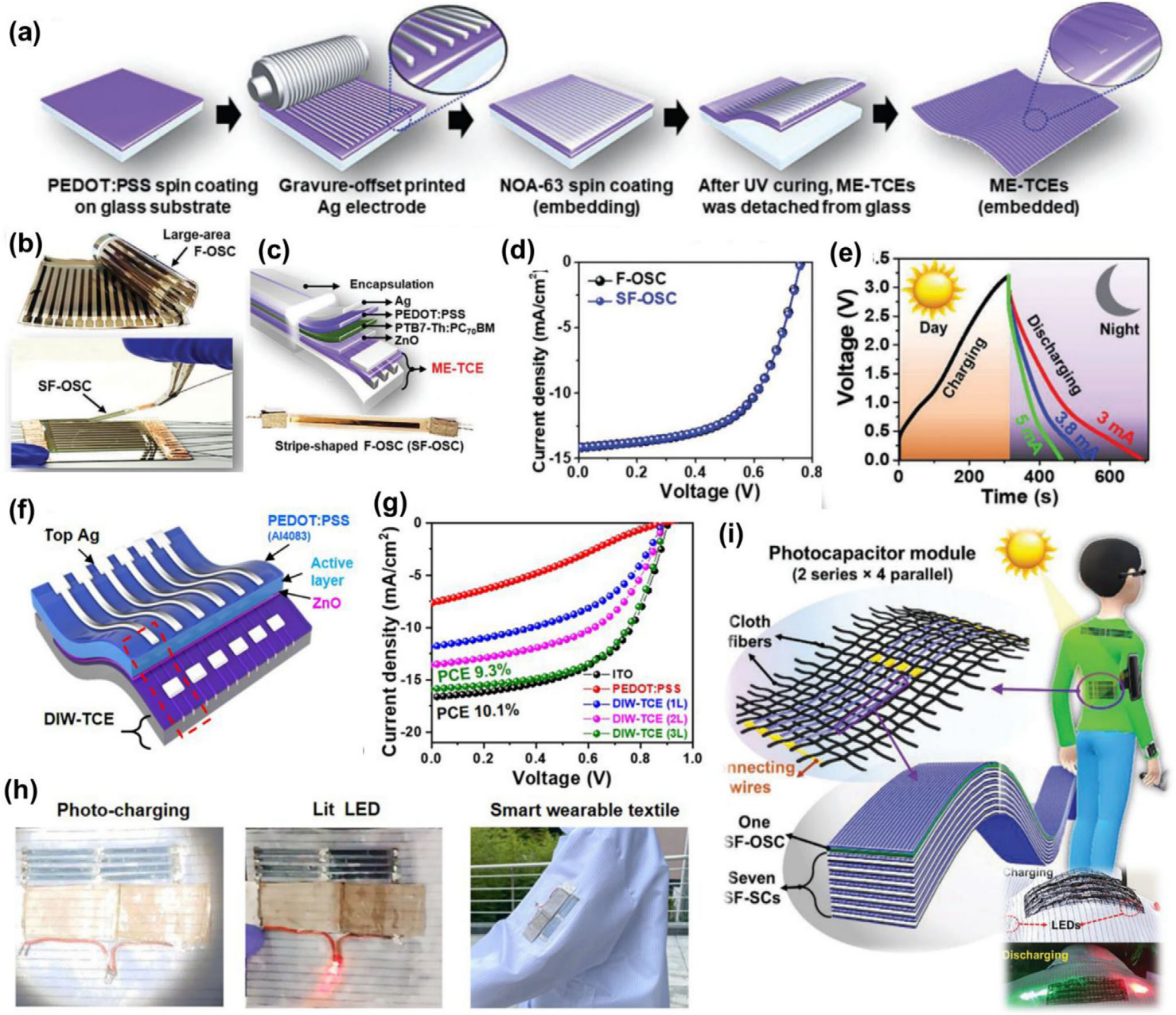
(a) A schematic shows how PEDOT:SS/Ag stripes ME‐TCEs are fabricated through a printing process. (b) A photograph demonstrates the slitting of large‐area F‐OSCs to create SF‐OSCs. (c) Diagram of a single stripe‐shaped F‐OSC device. (d) Comparison of *J*–*V* characteristics between large‐area F‐OSC and SF‐OSC. (e) The photo‐charge and galvanostatic‐discharge results of the photocapacitor module at different discharge rates. Reproduced with permission [120]. Copyright@ 2021, John Wiley and Sons; (f) Schematic of the flexible OSC device showing the arrangement of the top Ag and 3D Ag fiber. (g) *J*–*V* curves for OSCs with PEDOT:PSS and DIW‐TCEs (1L, 2L, 3L), compared with a reference ITO‐based rigid device. (h) Photographs display how a photo‐charged textile power module lights a commercial red LED, and its use as a portable power source integrated into fabric. Reproduced with permission [64]. Copyright 2023, Elsevier; (i) Flexible photocapacitor fibers in smart wearables, shown woven into a lab coat. Reproduced with permission [120]. Copyright@ 2021, John Wiley and Sons.

In another attempt, DIW‐TCEs with varying thicknesses (1L, 2L, and 3L) were utilized as electrodes to fabricate flexible OSCs. These OSCs employed an n‐i‐p stack: DIW‐TCE/ZnO/photoactive layer/PEDOT:PSS/Ag, as depicted in Figure 8f [64]. The current density–voltage (*J*–*V*) data (Figure 8g) indicate that flexible OSCs incorporating DIW‐TCEs outperform those with PEDOT:PSS electrodes, the latter achieving a maximum PCE of about 3.5%. In contrast, the flexible OSCs with 1L, 2L, and 3L DIW‐TCEs attained maximum PCEs of approximately 6.4%, 7.6%, and 9.3%, respectively. This enhanced photovoltaic performance is predominantly attributed to higher short‐circuit current density and fill factor, both of which increase systematically with the Ag fiber thickness. To further demonstrate their potential, a large‐area flexible supercapacitor was fabricated, integrated with the power module, and connected to a commercial LED on a textile substrate (see Figure 8h). When exposed to simulated sunlight for 5 min, the module charged the supercapacitor to 1.8 V, which subsequently powered the LED (Figure 8i).

Silver‐based TCEs produced via micro‐contact printing (µCP) have shown improved performance over commercially available ITO‐coated glass and plastic substrates. These Ag TCEs are also comparable to top‐performing Ag nanowire or nanonetwork (NN) electrodes used in organic photovoltaic (OPV) devices [202, 203, 204, 205]. The µCP enables the fabrication of transparent Ag grid electrodes by depositing perfluorinated polymers on silver‐attracting substrates and exposing them to silver vapor. Excess silver nanoparticles can be rinsed off with specialized solvents. OPV devices with PEDOT:PSS‐coated Ag grids achieve PCEs (10%), similar to those using commercial ITO glass electrodes, the industry standard [204]. An innovative approach to selectively depositing Cu and Ag TCEs that eliminates metal waste and harmful chemical etchants, ensuring a clean metal surface, an essential factor for advanced sensor and organic electronics applications. This method enables the fabrication of thin metal films with 6 million apertures per cm^2^, grids with approximately 1 µm lines, and apertures up to 10 cm in diameter [202]. This method is versatile and simple compared with the vacuum‐based, offering a budget‐friendly way to produce TCEs, with feature size and shape determined by the organofluorine patterning process.

Adding AgNWs or other noble metals can lower sheet resistance. Graphene quantum dots (GQDs) may further reduce *R_sheet_* owing to its tunable bandgap, high conductivity, and stability [206]. Currently, there is limited research on the use of GQDs in TCEs. For instance, Shin and colleagues incorporated GQDs and AgNWs into graphene, using this composite as a TCE in OPV [207]. The inclusion of GQDs enabled the electrode to maintain approximately 90% transparency within the visible spectrum. Additionally, the *R_sheet_* was substantially reduced: from 780 ± 50 Ω/□ for pristine graphene, to 123 ± 50 Ω/□ when GQDs and AgNWs were incorporated, and further down to 92 ± 6 Ω/□. Both the work function and sheet resistance of TCEs are critical factors influencing the FF in solar cells. Importantly, introducing 0.03 g/L of GQDs increased the work function of the GQDs/Ag nanowires/graphene TCE from 4.43 ± 0.02 to 4.53 ± 0.02 eV, thereby contributing to improved PCE. Additionally, some studies have employed graphene as both the anode and cathode in devices utilizing low‐bandgap polymers. A dry‐transfer method at room temperature was employed to place graphene onto the active layers [7, 208]. When these TCEs were combined with organic materials that primarily absorb in the ultraviolet and near‐infrared regions, the resulting devices exhibited optical transmittance as high as 61% in the visible spectrum and achieved power conversion efficiencies ranging from 2.8% to 4.1% [208].

Han et al. fabricated a composite TCE comprising PET/Ag‐PR/AgNWs via roll‐to‐roll printing, paving the way for efficient, large‐area, and flexible OSCs [209]. By applying a theoretical framework, they assessed electrical and optical losses in these monolithic grid‐based electrodes and developed guidelines for optimizing grid‐finger designs. The introduction of the equivalent resistance concept further simplified the design assessment process. For example, a flexible TCE incorporating 100‐micron‐wide silver (Ag) fingers spaced 5 mm apart on the AgNWs layer achieved an equivalent *R_sheet_* of 2 Ω/□ by maintaining the optical losses below 3%. Thus, large‐area flexible‐OPVs with active areas of 4 cm^2^ and 16 cm^2^ achieved efficiencies of 15.20% and 14.24%, respectively. There are still much work to be carried out on the degradation mechanisms and the stability of TCEs in solar cells under operating conditions, which may open roads to advances the photostability of OPVs.

### 4.2 Application of Printed TCEs in Photodetectors

Photodetectors are used to capture optical signals, including those from ambient light, and transform them into electrical outputs. These components are widely used in applications such as high‐speed optical communication systems, advanced imaging technologies, and wearable electronics [25, 210]. Advancing material systems, refining device structures, scaling production, and diversifying design strategies are all crucial for developing next‐generation photodetectors (PDs) without depending on traditional microfabrication methods. In response to these challenges, researchers have investigated various printing technologies for detector fabrication [210, 211, 212, 213].

ITO is commonly utilized as a TCE in optoelectronic devices due to its excellent transparency and low sheet resistance. However, ITO faces challenges such as limited indium supply and mechanical brittleness, raising concerns about future technological progress [214]. As an alternative, dielectric/metal/dielectric (DMD) structures have appeared, offering high transparency, low sheet resistance, and effective reflection of infrared radiation [215]. In DMD TCEs, an Ag layer is added between dielectric layers to further decrease sheet resistance and improve optical transmittance. Despite these improvements, most DMD designs still use dielectrics with low work functions, such as ZnO, WO_3_, TiO_2_, ITO, and SnO_2_, leading to relatively low barrier heights when paired with n‐type semiconductors [214, 215, 216, 217]. Yang et al. demonstrated a simple wet‐chemical lift‐off technique (Figure 9a) to develop the flexible TCEs consisting of a gold network, using an electrospun polymer fiber network as a mask. The resulting TCEs achieved a low *R_sheet_* of 5.18 Ω/□ at approximately 90% transparency [41]. Researchers constructed photodetectors from organolead halide perovskites using Au network electrodes. Figure 9b illustrates the relationship between photocurrent density and applied bias voltages (ranging from −0.9 to 0.9 V) for the CH_3_NH_3_PbI_3_ photodetector under both 500 nm monochromatic light and dark conditions. Under 500 nm illumination, the device showed a notable increase in photocurrent. The specific detectivity at zero bias remains nearly constant (approximately 10^1^ ^2^ Jones) across incident light intensities from 5 × 10^−9^ to 0.04 W/cm^2^. To evaluate flexibility, the CH_3_NH_3_PbI_3_ photodetector was constructed using two types of flexible TCEs: ITO/PET and Au network/PEN, which were then subjected to various bending radii. The Au network electrode‐based device showed a reduction in photocurrent at a smaller bending radius (about 5 mm), while the ITO/PET‐based device experienced this decrease at a larger radius (10 mm). This comparison indicates that the Au network electrode offers superior flexibility compared to commercial ITO electrodes. This enhanced flexibility is attributed to the integrity and high aspect ratio of the Au network, supporting the effectiveness of the wet‐chemical lift‐off technique. Additionally, increasing the Au network thickness does not significantly affect its optical transmission, suggesting that both conductivity and transparency can be improved by adjusting network thickness.

**FIGURE 9:**
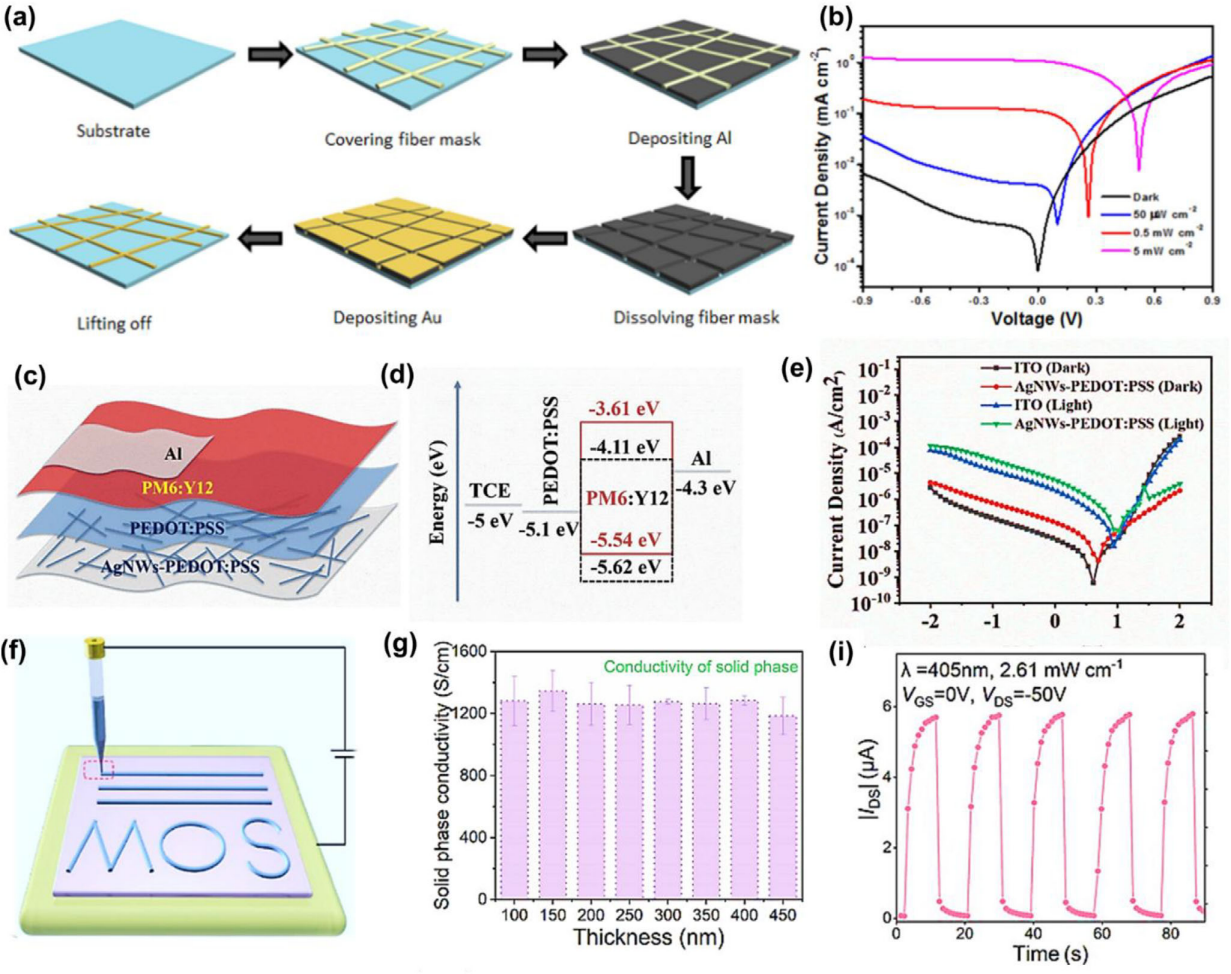
(a) Illustration of the fabrication steps for the patterned metal network TCEs. (b) Photocurrent density of the perovskite photodetector measured across a voltage range from −0.9 to 0.9 V. Reproduced with permission [41]. Copyright 2028, American Chemical Society. (c) Flexible OPD stack with AgNWs‐PEDOT:PSS as bottom TCE. (d) Energy level diagram, (e) *J*–*V* characteristics of the TCE and ITO‐based OPD in dark and white light‐illuminated conditions (1 mW cm^−2^). Reproduced with permission [27]. Copyright 2023, John Wiley and Sons. (f) Schematics of electrohydrodynamic jet printing PEDOT:PSS TCEs. (g) Conductivities with different thicknesses. (h) Photoswitching characteristics of phototransistor. Reproduced with permission [26]. Copyright 2024, American Chemical Society.

Further, Xiao et al. developed TCEs (Figure 9c) by controlling PEDOT:PSS aggregation and using EHD‐printed, narrow, highly conductive electrode lines (down to 5 µm, up to 1250 S/cm) [27]. Figure 9d shows the energy level diagram of the organic photodetector (OPD). Both OPDs were designed with an active area of 0.25 cm^2^. Figure 9e displays the (*J−V*) characteristics for OPDs using ITO and AgNWs‐PEDOT:PSS electrodes. The J_ph_ value of AgNWs‐PEDOT:PSS‐based OPD at −1.7 V bias was 100 µAcm−2, which was two times higher than the ITO‐based OPD. The AgNWs‐PEDOT: PSS hybrid electrode (Figure 9f) has been successfully utilized in OPD devices, demonstrating its potential for ultra‐flexible OPD applications [26]. These electrodes are used in organic field‐effect transistors and a transparent phototransistor (Figure 9g,h), both of which exhibit excellent light sensitivity (specific detectivity (D*) ∼ 2.860 × 10^14^ Jones) and potential for advanced optical imaging. This hybrid electrode achieves an average *R_sheet_* of 27 Ω/□ and optical transmittance ∼90% in the visible range, supporting its application in optoelectronic devices. Additionally, the AgNWs‐PEDOT:PSS hybrid TCE maintains a consistent change in the *R_sheet_* of less than 0.2 even after 50,000 cycles of continuous U‐folding at a radius of curvature as small as 0.1 mm. To further explore printing processing, a PEDOT:PSS:G:EC hybrid TCE was prepared using R‐2‐R printing, avoiding the use of strong acids [218]. These flexible electrodes show 78% transmittance, a *R_sheet_* of 13 Ω/□, and superior flexibility. They are ideal for flexible electronics, with organic solar cells using these electrodes reaching 9.4% efficiency. Hybrid ink (EG and PH1000) was spray‐coated onto preheated (90°C) PET or SiO_2_ substrates using an airbrush (0.15 mm nozzle, 3 bar nitrogen, 15 cm distance) [20]. Film thickness and transmittance were tuned by the number of spray cycles: 60 cycles yielded 11 nm (90% transmittance) and 100 cycles yielded 20 nm (80% transmittance). The films showed *R_sheet_* of 1200 Ω/□ and 500 Ω/□, and the 20 nm film reached a conductivity of ∼1000 S/cm, outperforming typical solution‐processed graphene films. Overall, TCE‐based architectures and scalable methods are key to developing flexible, high‐performance photodetectors for future optoelectronic applications, addressing the need for efficient, affordable devices.

### 4.3 Application of Printed TCEs in Light‐Emitting Diodes

#### 4.3.1 Organic Light‐Emitting Diodes (OLEDs)

The TCE, made up of metals, metal oxides, carbon, or composites, is explored in emerging electronics, such as OLEDs and PeLEDs, which are increasingly recognized as advanced display technologies [219, 220, 221, 222, 223]. They offer high brightness, broad viewing angles, rich color reproduction, fast refresh rates, and efficient power use [33, 224]. Additionally, their straightforward structure allows integration into ultrathin, flexible, foldable, and transparent screens [6, 29]. A mechanically resilient and highly flexible Ag mesh/ITO composite TCE, integrated with a micro‐lens array (µLA), was developed to improve light extraction in OLEDs [16]. Flexible OLEDs that use the Ag mesh/ITO composite transparent electrode exhibited superior mechanical durability compared to traditional ITO‐based devices, retaining almost all of their original luminance after 1000 bending cycles (radius ∼2 mm). Both experimental and theoretical studies showed notable enhancement in external quantum efficiency and angular emission profiles after incorporating the micro‐lens array (µLA), mainly due to the increased light scattering caused by the µLA. The combination of Ag mesh and µLA helps overcome the inherent brittleness of ITO while significantly improving light out‐coupling efficiency in flexible OLEDs, achieving an enhancement ratio of approximately 1.51. A new type of TCE was created by incorporating a PEDOT:PSS and silver fiber hybrid structure into a polymer substrate (see Figure 10a), thereby eliminating the need for ITO [29]. The hybrid‐fiber TCE, designed with a 150 µm pitch, demonstrates excellent mechanical flexibility and a low sheet resistance of 1.3 Ω/□. It consistently retains its electrical performance, even when subjected to bending at a radius as small as 0.5 mm. OLEDs fabricated with these electrodes perform comparably to those made with conventional ITO‐based TCEs. Additionally, when these OLEDs are slit into organic light‐emitting fibers (OLEFs), they deliver high luminance efficiency (up to 38.2 cd A^−1^) and EQE (up to 10.9%), surpassing previous records. Another essential requirement for OLEFs is uniform luminance. In this study, the uniformity of luminance across the fabricated OLEF, which has a total emission length of approximately 30 mm, was qualitatively assessed by measuring luminance at five distinct locations on the device, as illustrated in Figure 10b. Flexible OLEDs provide benefits such as high brightness, low energy consumption, and mechanical flexibility. Nevertheless, their performance can be constrained by the difference in refractive indices between air and the flexible substrate, which limits light outcoupling efficiency. To address this challenge, Ko and colleagues developed a durable oxide/metal/oxide (OMO) structured TCE, paired with an anti‐reflective (AR) film made from silica nanoparticles to improve light extraction in flexible OLEDs [106]. AR‐OMO structures were created on polyethylene terephthalate substrates using plasma‐enhanced chemical vapor deposition and magnetron sputtering (see Figure 10c). Tests showed that OLED devices using AR‐OMO TCEs had greater luminance efficiency (LE) and external quantum efficiency (EQE) compared to those with conventional OMO or indium tin oxide electrodes. This improvement is due to the AR film, which reduces waveguided light loss at the air‐substrate interface. The top‐performing AR‐OMO‐based flexible OLEDs recorded an LE of 12.3 cd/A and an EQE of 5.0% (Figure 10d). Furthermore, the AR‐OMO device retained full luminance even after being bent repeatedly to a 6 mm radius, demonstrating strong mechanical durability. These robust OLEFs tolerate substantial mechanical deformation and can be integrated into textile matrices for smart fabric applications. The scalable, vacuum‐free process used to fabricate these high‐performance OLEFs offers a promising approach for producing large‐area smart textiles.

**FIGURE 10:**
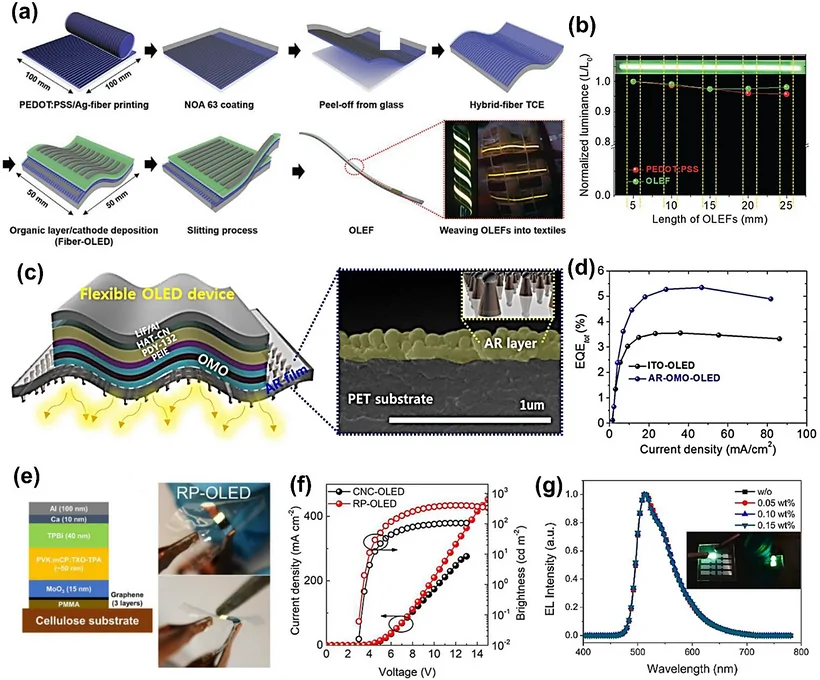
(a) Overview of the fabrication process for OLEFs. (b) Image of large‐area hybrid‐fiber TCEs (100 × 50 mm^2^), accompanied by microscope images of hybrid‐fiber TCEs with varying pitches. Reproduced with permission [29]. Copyright 2020, John Wiley and Sons. (c) Illustration of the device structure for flexible OLEDs utilizing different TCE substrates, along with their *EQE_tot_‐J* plots as measured with an integrating sphere. (d) Diagram of flexible OLED structure, including a bending test setup for devices using various hole‐injection materials on the prepared FTEs. Reproduced with permission [106]. Copyright 2021, Elsevier. (e) Illustration of OLED structure on cellulose‐based substrates, with Photographs of RP‐OLED, under bending state. (f) Current density and brightness of these OLEDs as a function of applied voltage. Reproduced with permission [220]. Copyright 2025, Royal Society of Chemistry. (g) Diagram depicting the crosslinking reaction between PEIE and PSS at the PEIE/PEDOT:PSS interface. Shown are current density‐voltage‐luminance and electroluminescent spectra at 1000 cd/m^2^ (Inset: Images of flexible OLEDs when activated). Reproduced with permission [225]. Copyright 2021, Elsevier.

Recently, Kim et al. developed a technique to pattern metal‐mesh‐type TCE using desorption processes in solution‐processed PVDF–HFP, followed by transfer printing. This approach was further enhanced by thermally evaporating silver onto the patterned PVDF–HFP, yielding metal‐mesh electrodes with high transparency and low resistance. This streamlined technique integrates easily with sensitive organic materials, eliminating the need for lamination or solvent treatment [226]. TCEs with AgNWs and AZO were combined via a solution‐based method to form a flexible, transparent conductive film (TCF). Spinning and annealing at 120°C fuse the AgNW network, enhancing both adhesion and smoothness. The fabricated TCE achieves a *R_sheet_* of 12.9 Ω/□ and a transmittance of 92.1% at 550 nm, making it suitable for use as an OLED anode. When incorporated into a flexible green OLED, this TCF delivers a luminous intensity of 10 060 cd/m^2^ and a current efficiency of 6.43 cd/A, highlighting its promise in flexible electronic applications [227]. OLEDs were fabricated using a printed copper grid with line widths under 3 µm. The addition of a thick, doped hole injection layer significantly reduced electrical shorts and enabled the production of high‐quality OLEDs with minimal leakage current. Through advanced printing methods, achieving a fine pitch of 5 µm and a line width as narrow as 250 nm, the luminance performance of the Cu grid OLEDs is comparable to that of traditional ITO‐based OLEDs [223].

Further, Kulyk and colleagues improved OLED devices by utilizing graphene anodes on cellulose nanocrystal membranes and cellulose‐based rolling papers [220]. The use of these clear, biodegradable materials offers a sustainable alternative to traditional synthetic substrates for flexible OLEDs. By stacking layers of graphene and adding MoO_3_, the electrical conductivity of the anode was significantly enhanced (Figure 10e). This process enabled the creation of third‐generation, solution‐processed OLEDs that achieved EQE as high as 0.34% and maximum brightness near 400 cd/m^2^ (see Figure 10f). Applying a PEIE layer to PEDOT:PSS electrodes improves their flexibility, stability, and current efficiency in OLEDs by 2.7 times, thanks to interfacial crosslinking (Figure 10g) [225]. Looking ahead, the advances in TCE technologies, such as flexible hybrid structures, novel anti‐reflective coatings, and innovative patterning methods, are poised to revolutionize the next generation of flexible and wearable electronics. These developments pave the way for highly efficient, durable, and scalable OLED displays and smart textiles, opening exciting prospects for large‐area, foldable, and integrated electronic devices in both consumer and industrial applications.

#### 4.3.2 Perovskite Light‐Emitting Diodes (PeLEDs)

Perovskite quantum dots (PeQDs) possess notable properties such as high quantum efficiency, sharp emission profiles, adjustable emission wavelengths, and the ability to be processed via solution‐based methods. These characteristics have facilitated advancements in Perovskite light‐emitting diodes (PeLEDs), resulting in devices with superior color purity, enhanced brightness, and high EQE. With further miniaturization, PeLEDs could become viable alternatives to traditional semiconductor micro‐LEDs, benefiting from simpler fabrication, lower costs, and higher yields [228, 229]. However, most PeLEDs are currently made by spin coating, a method that limits precise control over pixel size and results in significant material waste [230]. Zhao et al. fabricated PeLEDs (Figure 11a) on elastomer substrates, utilizing a scalable inkjet printing method to pattern all device layers from the bottom anode to the top cathode [101]. In contrast to traditional microfabrication techniques, this printing approach significantly shortens fabrication time, reducing it from over 5 h to less than 25 min, a more than 10‐fold improvement. The fully printed PeLEDs exhibit a distinctive four‐layer design comprising a bottom electrode, a perovskite emissive layer, a buffer layer, and a top electrode, eliminating the need for separate electron or hole transport layers (see Figure 11b). Figure 11c displays optical micrographs of printed PeLEDs as seen from both the AgNW cathode (top) and PEDOT:PSS anode (bottom) sides. The active light‐emitting region, defined by the 500 × 500 µm^2^ overlap between the PEDOT:PSS/PEO bottom electrode and the AgNW top electrode, becomes visible when a bias voltage is applied. In Figure 11d, the PeLED emits green light under a forward bias of 4.5 V, producing a luminance intensity of approximately 4200 cd/m^2^. Figure 11e shows that increasing the number of printing passes from 5 to 40 results in a sharp reduction in *R_sheet_*, from 6900 to 2.2 Ω/□. As depicted in Figure 11f, the three *J–V* curves are almost identical after 100 cycles, with no significant differences observed. Figure 11g presents L–V curves that remain consistent, showing only a minor decrease in luminance: from 8885 cd/m^2^ (fresh device) to 8250 cd/m^2^ (after 100 bending cycles) at 9.0 V. The inset of Figure 11g illustrates that even when the PeLED is bent to a concave shape with a 3.5 mm radius, it continues to emit stable green light. When printed in ambient conditions, the flexible PeLEDs display showed a turn‐on voltage of 3.46 V, reach a peak luminance of 10 227 cd/m^2^, and attain a maximum current efficiency of 2.01 cd/A.

**FIGURE 11:**
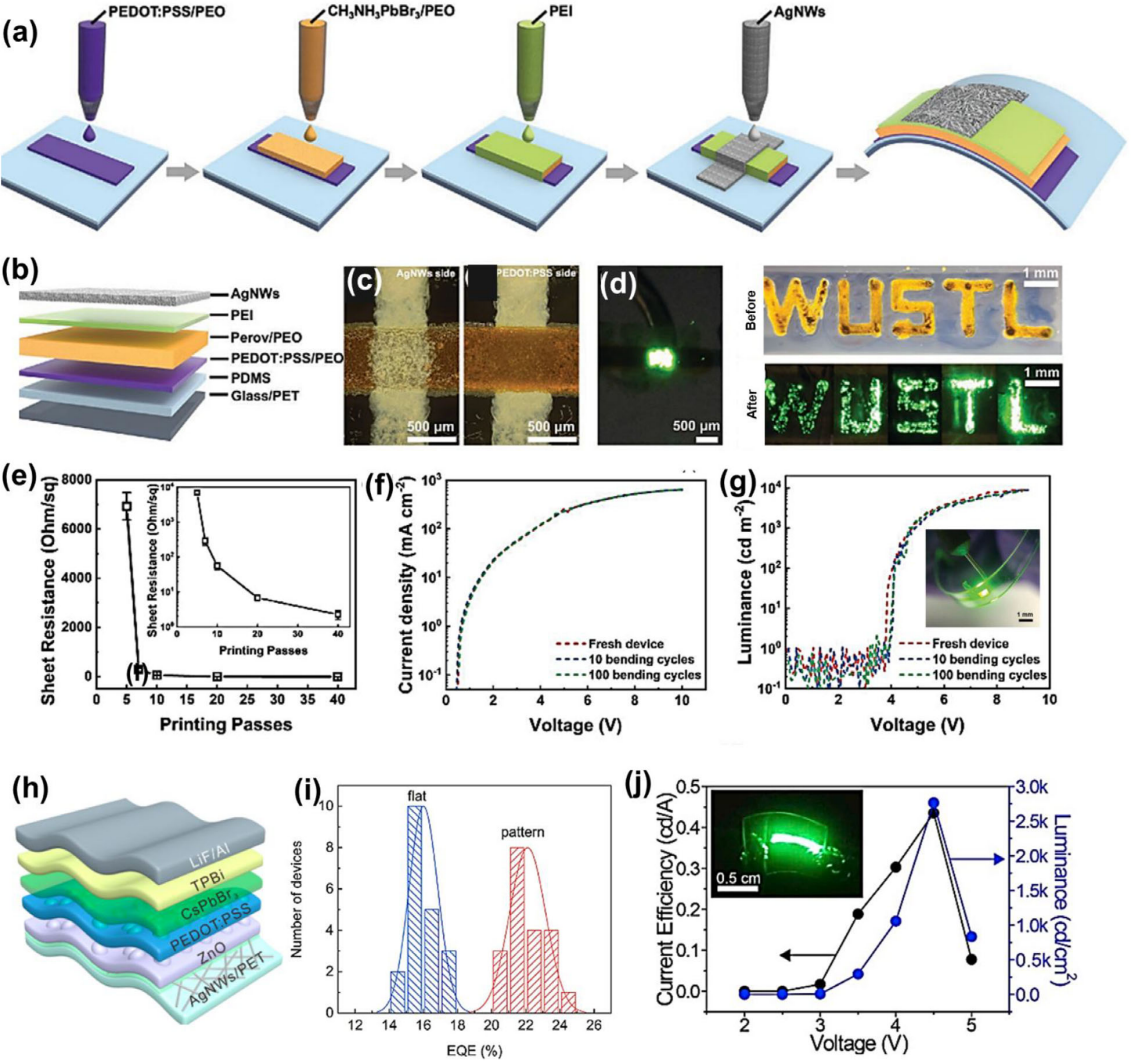
(a) Schematics of the fabrication process of all‐inkjet‐printed flexible PeLEDs. (b) Stacked structure of the printed PeLED device. (c) Optical images of a PeLED and (d) an illuminated PeLED under 4.5 V, and a prototype with “WUSTL” characters: before and after being powered at 3.5 V. (e) The R_sheet_ vs. No. of printing passes, with a semi‐logarithmic (inset). The flexible PeLEDs underwent bending tests. (f) Current density and (g) luminance as functions of voltage with bending cycles. (*Inset*) PeLED on flexible PDMS, bent to a 3.5 mm curvature. Reproduced with permission [101]. Copyright 2021, John Wiley and Sons. (h) Schematic of the device architecture on flexible PET substrate featuring a composite AgNW electrode. (i) Histograms comparing peak EQEs for PeLEDs with flat and patterned designs. Reproduced with permission [231]. Copyright 2020, American Chemical Society. (j) Flashlight irradiation demonstrates current efficiency and luminance in flexible PeLEDs. Reproduced with permission [232]. Copyright 2019, Elsevier.

Further, achieving micro‐PeLEDs with sizes of just a few tens of micrometers while maintaining uniform, intense luminescence remains a significant challenge [233]. Micro‐PeLED arrays emitting red, green, and blue light, with pixel dimensions down to 45 µm, were fabricated by depositing a PeQD emissive layer through in situ inkjet printing. The device architecture is composed of ITO/(PEDOT:PSS)/PVK/SDS/PeQD/TPBi/LiF/Al. These arrays demonstrate external quantum efficiencies of 0.832% (red), 0.419% (green), and 0.052% (blue), along with a resolution of 210 PPI and a color gamut reaching 135% of the NTSC standard. Further, a flexible PeLED (Figure 11h), engineered with optimized interfaces and an AgNW electrode on a patterned plastic substrate, shows enhanced crystallization, reduced defects, and improved light outcoupling [231]. This design achieves a 24.5% external quantum efficiency for green‐emitting CsPbBr_3_ perovskite, as shown in Figure 11i. A recent study developed a new method to boost the performance of flexible PeLEDs using a transparent, ITO‐free WO_3_/Au/WO_3_ electrode [234]. The additive P123 in the perovskite solution accelerates crystallization and improves crystal quality, resulting in small, uniform grains and fewer recombination centers. Thus, the green PeLEDs showed high luminance (10 200 cd/m^2^), an EQE of 14.45%. Flexible devices also maintained strong performance after repeated bending, retaining over half their luminance and most of their current efficiency after 1000 cycles. In another attempt, Jung et al. achieved rapid recrystallization of the perovskite using flash light annealing (FLA) [232]. Notably, even with the relatively uneven surface of commercially available PEN substrates and ITO films of moderate conductance, the flexible PeLEDs fabricated via FLA demonstrated significantly improved performance (Figure 11j), showing a 252% increase in current efficiency and 409% higher luminance compared to devices made using traditional thermal annealing. This method provides a scalable, cost‐efficient pathway to fabricate high‐performance, flexible PeLEDs suitable for diverse applications in displays and wearable electronics. Ongoing improvements in fabrication methods, like scalable inkjet printing and flexible electrodes, will likely boost PeLED performance and production. These advances could speed up the use of perovskite devices in new displays, wearables, and other technologies, supporting wider commercial adoption.

### 4.4 Applications for Printed TCEs in Energy Storage and Electrochromic Energy Storage Devices

Transparent conducting electrodes or films (TCEs/TCFs) enable devices that store charge while remaining optically transparent and frequently mechanically flexible, offering a feasible foundation for next‐generation energy storage [108]. Supercapacitors, thin batteries, and electrochromic energy‐storage systems can now be directly incorporated into wearable platforms, smart windows, and displays without compromising light transmission or aesthetics [235]. Beyond transparency, the TCE/TCF architecture provides consistent current collection at low sheet resistance, supporting multifunctional designs such as energy storage with visual state indication (electrochromism), heating, or sensing, and assisting in maintaining performance under bending. In flexible optoelectronic devices, low‐sheet‐resistance TCEs play a vital role in their fabrication. In our recent work, 3D fiber‐shaped supercapacitors (SCs) utilizing MXene‐PEDOT:PSS as the TCE (Figure 12a), employing a three‐dimensional direct‐ink‐writing method [158]. The integration of a silver current collector inside the electrode promotes more efficient charge movement throughout the fiber‐shaped device. This structural innovation overcomes challenges tied to electrode thickness and length that typically hinder capacitance in fiber‐based supercapacitors. The resulting one‐meter‐long (Figure 12b), fully printed 3D‐SC offers low charge transfer resistance and achieves impressive electrochemical performance, with areal capacitance of 1.062 F/cm^2^, gravimetric capacitance of 185.9 F/g, and areal energy density of 94.41 µWh/cm^2^ at a power density of 1142 µW/cm^2^. In another study from our lab, Ovhal et al. used the D‐bar coating method to fabricate PEDOT:PSS‐based large‐area (9 and 64 cm^2^) TCEs for application in transparent, solid‐state, flexible SC (shown in Figure 12c,d) [75]. The TCE exhibited a high Ca of 4.7 mF/cm^2^ (as evident from, about 35% higher than that for spin‐coated TCEs. The large‐area (9 and 64 cm^2^) symmetric device with this TCE and PVA/LiCl had an optical transmittance of >65% while still delivering a C_a_ of 3.2 mF/cm^2^ and 2.9 mF/cm^2^, respectively (Figure 12e).

**FIGURE 12:**
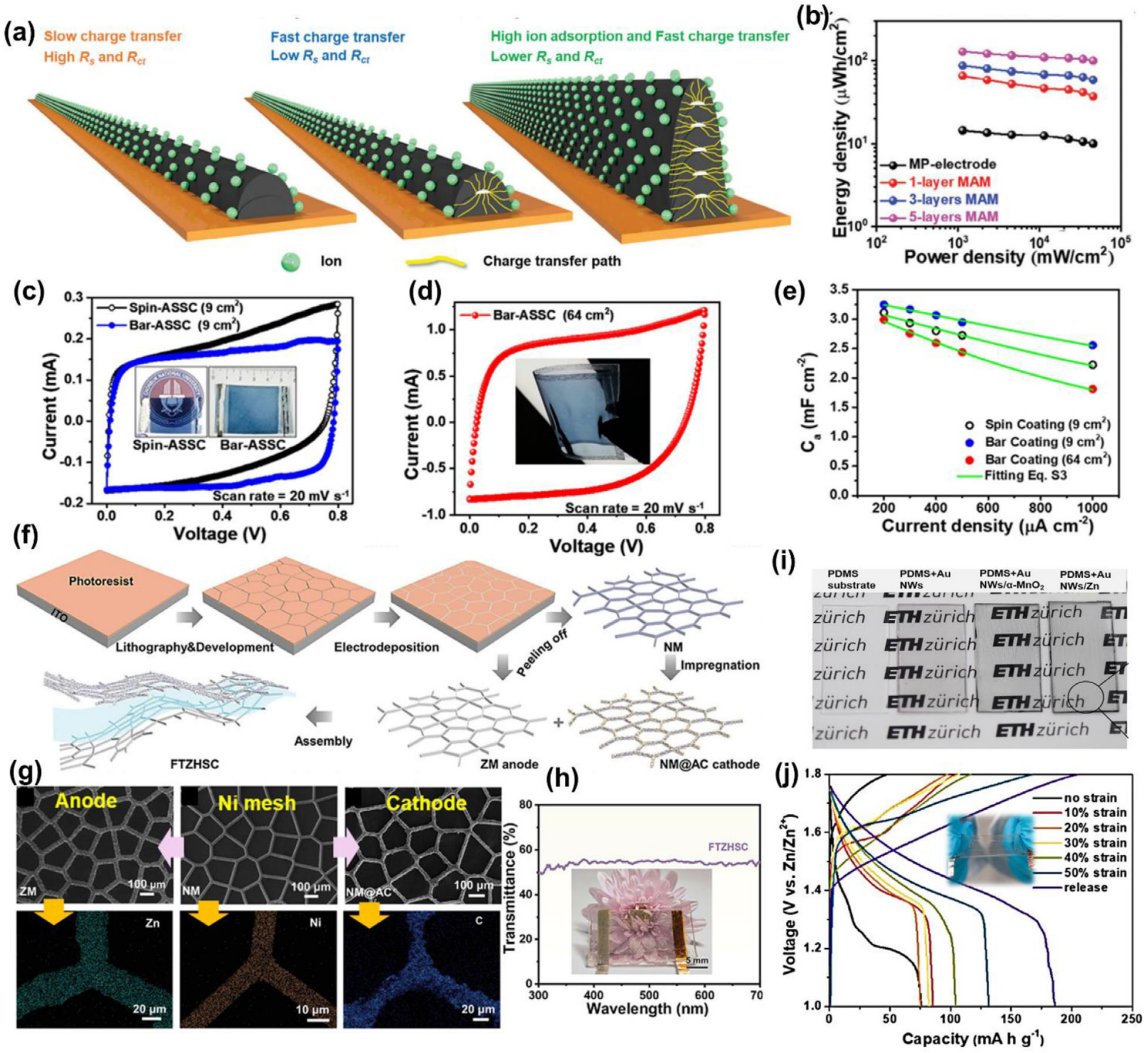
(a) Schematic illustration of the efficient charge transport mechanism in multilayer metal‐embedded electrodes. (b) Ragone plot of different multilayer Ag‐embedded MXene/PEDOT electrodes compared to non‐embedded ones. Reproduced with permission [158]. Copyright 2024, John Wiley and Sons. CV curves of the spin‐coated ASSC and bar‐coated ASSC with area of (c) ∼9 and (d) ∼64 cm^2^ bar‐coated ASSC, with the inset containing the digital images of the real devices. (e) Variation of C_a_ with current. Reproduced with permission [75]. Copyright 2020, Elsevier. (f) Schematic illustration of flexible anode and cathode TCE fabrication, (g) FESEM images of the developed cathodes and anodes. (h) Plot showing the transmittance of the device with the inset showing the real device. Reproduced with permission [78]. Copyright 2024, John Wiley and Sons. (i) Digital images of the cathode‐ and anode‐coated PDMS substrate, bare PDMS substrate, and that of the fabricated full cell, (j) GCD profiles of the transparent battery at various strains of 0–50%. Reproduced with permission [236]. Copyright 2021, American Chemical Society.

Figure 12a shows the effect of multilayer Ag electrode addition inside MXene/PEDOT electrode, leading to higher performance as a supercapacitor electrode. In this regard, our lab (Jin et al.) fabricated spin‐coated PEDOT:PSS and Ag‐embedded ME‐TCE through gravure‐offset printing, with good transmittance and low sheet resistance for use in a photocapacitor module [120]. In the study, the ME‐TCE served as the top electrode for the OSC of the photocapacitor module and, together with a flexible supercapacitor using the same TCE with rGO added to PEDOT:PSS, achieved a high‐power conversion efficiency of 6.43%. Zhao et al. fabricated a 2D‐conducting Cu_3_(HHTP)_2_ MOF layer with excellent electrochemical and optoelectronic properties over an ITO/PET electrode via layer‐by‐layer assembly for use as a flexible TCE [237]. In their study, they found that the flexible TCE fabricated using 15 coating cycles had a transmittance of 82.2% at a sheet resistance of 49.1 Ω/□ and showed a high C_a_ of 1700 µF/cm^2^. The symmetric device fabricated with this flexible TCE had a transmittance of 62.1% with a high C_a_ of 939.2 µF/cm^2^ at 7 µA/cm^2^ with good cyclic stability. In their other work, a coated Ni_3_(HITP)_2_ conducting MOF thin film via stamping on the solution over ITO/PET as the active electrode, and with a PEDOT: PSS‐based asymmetric device resulted in an increased C_a_ of 1.06 mF/cm^2^ at 3 µA/cm^2^ at similar transmittance [238]. Soram et al. fabricated a highly transparent asymmetric supercapacitor with a high C_a_ of 2.42 mF/cm^2^ at 0.02 mA/cm^2^ using a Ni mesh TCE as the current collector [239]. In the study, a cracked silica layer on the substrate was used as a mask for Ni deposition via E‐beam, followed by electrochemical deposition of the positive and negative electrodes. Metal mesh has also been developed by lithographically patterning of the photoresist layer over ITO, followed by electrodeposition, for use as TCE [240]. The Ni mesh (*R* = 0.7 Ω/□, *T* = 92.1%) synthesized by this process can be transferred to a PDMS substrate and used to develop electrodes for transparent SC through electrodeposition, leading to an asymmetric SC with transmittance of 67.5% with a high areal energy density of 8.0 µWh/cm^2^ and good stability. Jena et al. developed wafer‐sized Ag NW/PDMS TCE with metal‐assisted chemical etching and explored it for flexible, electrochromic zinc ion battery applications [241]. Zhang et al. used a similar lithographic process, depositing a photoresist layer on the ITO substrate, followed by electrodeposition of a Ni electrode and peeling off the photoresist to use as a TCE [78]. The detailed TCE fabrication process is illustrated in Figure 12f. Further, they used it to prepare a Zn anode and an AC cathode (FESEM and elemental mapping images are depicted in Figure 12g to fabricate a flexible and transparent Zn‐ion capacitor that had a high areal energy density of 31.69 µWh/cm^2^ and a high‐power density of 512 µW/cm^2^ while still maintaining an optical transmittance of 55.04% (Figure 12h), showing its capability for application in a transparent power supply. Kim et al. utilized the dewetting phenomenon of AgNW IPA dispersion on an OTS‐coated substrate to create web‐like AgNW electrodes with a high degree of transparency (90%) and good conductivity [242]. They used the meniscus‐dragging deposition technique to coat Ag NW webs on the OTS substrate, followed by coating Au over them via chemical deposition. Finally, they prepared an MnO_2_‐coated Ag‐Au web via electrodeposition, and a symmetric device using this TCE had a C_a_ of 3.8 mF/cm^2^ at a transparency of 90.4%. It was also shown that the MnO_2_ loading can be varied, resulting in capacitances ranging from 591.4 F/g at 5 µg/cm^2^ to 284.8 F/g at 60 µg/cm^2^, at similar transparencies. MXene as a TCE material has also been fabricated via different processes, such as the work of Guo et al., who used a slot‐die to deposit aqueous MXene inks to fabricate transparent SCs over PET [243]. The MXene‐based TCE was laser‐scribed to prepare an interdigitated micro‐SC, which had a C_a_ of 254 and 427 µF/cm^2^ at 20 mV/s at 0% and 60% transmittance, respectively. Wen et al. used inkjet printing for the fabrication of MXene‐based TCEs with a sheet resistance of 1.66 ± 0.16 MΩ/□ to 1.47 ± 0.1 kΩ/□ at the transmissivity of 87%–24% [244]. The MXene TCE had a C_a_ of 887.5 µF/cm^2^ at 24% transmittance, while the asymmetric SC fabricated with this TCE had a C_a_ of 192 µF/cm^2^ at still higher transmittance, better than the SWCNT‐based TCE. Vanadium MXene has also been printed by an inkjet printer over a variety of substrates by Ji et al. to fabricate TCEs with transmittance ranging from 42% to 87% for various mass loadings and sheet resistance of 2.8 ± 0.1 to 24.3 ± 0.9 kΩ, respectively [245]. These V_2_CT*_x_* films showed a C_a_ of 531.3, 933.6, 1807.5, 3133.1, and 5787.0 µF/cm^2^ at 5 mV/s, with the symmetric SC showing a C_a_ of 23.4 µF/cm^2^ at 5 mV/s. Oxides have also been used to fabricate flexible, transparent TCE. Wang et al. fabricated a PEDOT‐coated V_2_O_5_ NW and a graphene‐based, flexible TCE via filtration, followed by film transfer over an AgNW film on a flexible substrate, yielding a high Ca of 22.4 mF/cm2 at 70% transparency with good flexibility [246]. The symmetric SC fabricated using this electrode had a transparency of 55% and a high energy density of 0.18 µWh/cm^2^ at a power density of 11 µW/cm^2^. Huang et al. used Na_x_WO_3_ NW‐based TCE to fabricate a solid‐state, flexible, transparent supercapacitor with solar‐thermal‐enhanced performance, using blade‐coated Ag NWs and PEDOT:PSS as the conducting backbone and binder, respectively [247]. The performance of the symmetric SC was enhanced by the heat generated from NIR solar illumination during the photothermal conversion of Na_x_WO_3_. The device also showed good stability, indicating its potential as a flexible, transparent energy storage device with enhanced solar thermal performance. A similar study was conducted by Hsu et al. for flexible TCEs, using RuO_2_‐decorated Cs_x_WO_3_ composite nanorods as the negative electrode [248]. Similar high photothermal conversion and performance enhancement were observed for the electrode, including high flexibility and stability. Chen et al. used the spray‐coating technique to develop a carbon dot‐doped PANI as an active electrode for an electrochromic supercapacitor [249]. A V_2_O_5_@PEDOT‐based transparent electrode was used as the counter‐ion storage electrode, leading to high energy/power density (51.87 µW/cm^2^/4.02 µWh/cm^2^), fast switching times (tc = 0.75 s, tb = 2.61 s), and outstanding coloring efficiency (221.6 cm^2^/C).

Transparent electrolytes and thin active layers, combined with TCE/TCF architectures, enable transparent batteries to deliver useful energy while preserving mechanical durability and optical clarity. Liu et al. used spray coating of Au NWs and active electrodes over a PDMS mould as a TCE to construct a transparent, flexible, and stretchable Zinc‐ion battery (ZIB) with a high transmittance of 72% without strain and 64.7% at 50% strain [236]. For the fabrication of the TCE, AuNWs were first spray‐coated onto a hexagonal PDMS mould, and the excess was removed using Scotch tape. After oxygen plasma treatment of the substrates, α‐MnO_2_ and Zn nanoparticles were coated as active electrodes. Digital images of the different TCEs along with the PDMS substrate are presented in Figure 12i, showing their optical transparency. With a polyacrylamide‐based ZnSO_4_ and MnSO_4_ gel electrolyte, the stretchable, transparent ZIB (digital image presented in the inset of Figure 12j) delivered capacities of 186.3 and 136.2 mAh/g at 0% and under 50% strain (Figure 12j), respectively, at 1C. Nie et al. fabricated a freestanding, transparent, and stretchable serpentine Ni micromesh via photolithography and electrodeposition [250]. After photolithography of the photoresist layer over ITO and Ni electrodeposition, the photoresist layer is removed, and the Ni layer is peeled, and then NiCoP and Zn were electrodeposited for the cathode and anode, respectively. The electrodes had high optical transmittance of 81.4% and 70.2% for the cathode and anode, respectively, while the Zinc‐ion battery (ZIB) fabricated with them delivered a specific capacity of 5.7 µAh/cm^2^ at a current density of 0.6 mA/cm^2^, with an optical transmittance of 54%. Chen et al. fabricated a patterned 3D‐Ni mesh similar to the above study, with higher optoelectronic properties (T = 89.59% and Rs = 0.23 Ω/□) and super‐flexibility [251]. The 3D Ni mesh served as the TCE for the Cu(OH)_2_@NiCo BH cathode and the Cu@Zn anode in the fabrication of a solid‐state NiCo‐Zn alkaline battery, exhibiting energy and power densities of 35.89 µWh/cm^2^ and 2000.26 µW/cm^2,^ respectively, while maintaining a transmittance of 54.34%.

Qiu et al. used inkjet printing to fabricate a heterogeneous tungsten oxide nanowire ink for a Zn anode‐based electrochromic device with rapid color‐changing kinetics [252]. In the study, they used an MXene‐based 3D mesh as a substrate modifier before inkjet printing the tungsten oxide layer, resulting in TCE with optical transmission comparable to that of a non‐patterned substrate while also shortening the response time. A Zn‐based electrochromic device fabricated using a kitten‐patterned electrode and Zn anode maintained a high energy capacity retention of 98.5% and a stable coloration contrast of 97.7% over 14 days. Further, the non‐patterned device had an optical transparency of ∼70% and retained 50.44% of color contrast even after 1000 cycles. Another study by Dadkhah et al. used spray coating to fabricate TCE from 0D, 1D, and 2D tungsten trioxide nanostructures for use in an electrochromic device for smart window applications [253]. The study concluded that 2D WO_3_ TCEs had the highest transparency (∼70%) and the highest Li^+^ intercalation efficiency, achieving a high capacitance of 25.1 mF/cm^2^, an optical reflectance of 42.63%, and a shorter bleaching time of 3.79 s. The 2D WO_3_ structure also had a high coloration efficiency of 89.29 cm^2^/C at 700 nm, compared to 22 cm^2^/C for 1D and 8 cm^2^/C for OD, thus providing design rules for electrochromic coatings. Dutta et al. used a WO_3_.H_2_O/rGO nanocomposite ink for the fabrication of a low‐cost hybrid TCE with a large area (25 cm^2^) [254]. This nanocomposite was spray‐coated onto a 5 cm × 5 cm ITO/Al mesh TCE, yielding a blue‐to‐colorless electrochromic device with bleaching and coloration times of 0.43 and 2.16 s, respectively, making it one of the fastest‐operating devices. Compared with the transmittance modulation of ∼13% at 620 nm and 21% at 700 nm, the ITO/AL mesh electrode showed higher optical modulations of 16% and 24% at 620 and 700 nm, respectively. Hu et al. used a combined embedded Cu@Ag current collector over ITO and an IJP electrochromic polymer layer to develop large‐area, flexible electrochromic devices [255]. The embedded Cu@Ag current collector led to a sheet resistance of 0.2 Ω/□, compared to 35 Ω/□ for ITO alone. The IJP device had a fast response of ≤1.2/1.9 s for coloration/bleaching, with a 10 cm × 10 cm device fabricated using this technique showing good color uniformity within one second of operation. All things considered, the most recent examples show that printed TCE/TCF have evolved from “transparent current collectors” into practical, device‐enabling architectures that enable transparent batteries, transparent supercapacitors, and electrochromic energy storage across a range of material systems (metal meshes/nanowires, MXenes, MOFs, carbon networks, and oxide–polymer hybrids).

Table 2 provides an overview of key printing methods for fabricating TCEs, such as inkjet, electrohydrodynamic, gravure, screen, 3D printing, blade coating, bar coating, spray printing, and layer‐by‐layer printing techniques, and their application in different devices and their performance.

**TABLE 2:** Summary of characteristics and innovative applications of various TCEs fabricated using advanced printing techniques.

No.	Printing method	Material	R_sheet_ (Ω/□)	T_550 nm_ (%)	Application	Performance	Refs.
1.	Inkjet	Ag‐grid/PEDOT:PSS	10.3	73	Organic‐PV	PCE ∼ 2.5%	[126]
2.	Inkjet	PEDOT:PSS	—	—	OLED	CE ∼ 9.69 ± 0.37 cd/A	[127]
3.	Inkjet	PEDOT:PSS/ Ag Grid	12	89	Supercapacitor	*C_SC_* ∼ 4.72 mF/cm^2^	[128]
4.	Inkjet	PEDOT:PSS	29.5	97	Biological ‐PV	P_D_ ∼ 0.42 × 10^−3^ mW/cm^2^	[131]
5.	Inkjet	AgNPs	9.56	64.5	OLED	V_D_ ∼ 6V	[135]
6.	DIW	CB/Ag/CB	—	>80	Supercapacitor	C_S_ – 363 mF/g	[108]
7.	DIW	AgNWs/ PEDOT:PSS	23	86	Electric heater	85°C at 6 V	[123]
8.	DIW	AgNWs	25.3	98.6	Transparent heater	110°C at 6 V	[160]
9.	DIW	α‐ITO/Ag	<1	85	ST‐Perovskite‐PV	PCE ∼ 16.26%	[65]
10.	DIW	Ag/PEDOT:PSS	<4	80	Organic‐PV	PCE ∼ 9.3%	[64]
11.	Bar	AgNW/ PEDOT:PSS	25	85	Bio‐signal Sensing	50% strain and 500 cycles	[176]
12.	Bar	HPMs‐AgNW	17.1	94.7	Wearable electronics	—	[177]
13.	Bar	PEDOT:PSS	20 ± 5	>80	Supercapacitor	C_a_ ∼ 2.9 mF/cm^2^(∼64 cm^2^)	[75]
14.	Bar	Ag/MXene	9.4	93	Transparent EMI	SE ∼ 30.1 dB	[181]
15.	Gravure	Ag/ PEDOT:PSS	11	49	Photocapacitor	𝜂 ∼ 4.94%	[120]
16.	ROP	AgNW	34	84	OLED	Yellow (3 to 15 V)	[132]
17.	Gravure	PEDOT:PSS/Ag‐Fiber	1.3	59	OLED	EQE ∼ 11.3 ± 0.2%	[29]
18.	Gravure	CuNWs	31.6	79	Flexible LED	Stable (B_r_ ∼ 1 mm)	[154]
19.	Screen	Ag Microgrids/BN	2.9	65	Transparent heaters	74°C at 2.5 V	[156]
20.	Screen	Ag NWs	7.3	79.6	EL Display	L ∼ 25.7 cd/m^2^	[157]
21.	Blade	Au micromesh	80	86	OLED	EQE ∼ 3.6%	[162]
22.	Blade	Ag lines	3.1	78.2	Transparent heater	90°C at 1.5 V	[163]
23.	Blade	MXene	—	83.4	Heater	77°C at 5 V	[164]
24.	Blade	AgNWs:SnO_2_	14	87.6	Organic‐PV	PCE∼ 13.5%	[107]
25.	Spray	AgNW	49.5	66.7	Perovskite‐PV	PCE∼ 11.6%	[167]
26.	Spray	PEDOT:PSS	70	80	Flexible Touch Panel	Stable (2.43 mW/cm^2^ & 90% RH)	[168]
27.	Spray	AgNW	—	80	EC display	𝜂_c_ ∼102 cm^2^/C at 400 nm	[169]
28.	Spray	SnO_2_	—	95.6	Printed Perovskite‐PV	PCE∼ 16.9%	[102]
29.	Spray	CuNW‐GNs	81.42	87	Flexible connector	Stable (B_r_ ∼1–3 mm); 1000 cycles	[173]
30.	Spray	AuNWs	30	72.6	Zinc Ion Battery	C∼176.5 mA h/g after 120 cycles	[236]

**Footnote**: R_sheet_ – sheet resistance; T_550 nm_‐ transmittance at wavelength 550 nm; PV‐ Photovoltaics; PCE‐ power conversion efficiency; CE‐ current efficiency; C_SC_ – specific capacitance of the electrode; P_D –_ power density; V_D_ – driving voltage; C_S_ – specific capacitance; C_a_ – areal capacitance; SE – shielding effectiveness; 𝜂 – photoelectric‐conversion‐and‐storage efficiency; ROP – reverse offset printing; EL – electroluminescent; EQE – external quantum efficiency; B_r_ – bending radius; L – luminance; RH – relative humidity; EC – electrochromic; 𝜂_c_ – coloration efficiency; C‐ capacity.

### 4.5 Applications for Printed TCEs in Sensors

Sensors are a key part of modern electronics because they allow devices to detect changes in their surroundings and convert them into useful electrical signals. Using TCEs makes it possible to build sensors that remain optically transparent while still providing reliable electrical conduction and signal detection, which is especially useful for wearable, biomedical, and human–machine‐interface devices. Printing methods facilitate low‐cost patterned electrodes on flexible substrates using AgNWs, conductive polymers, carbon materials, MXenes, and their hybrids, which are increasingly being used in pressure, strain, touch, temperature, chemical, and bio‐signal sensors. For instance, Gilshtein et al. fabricated ITO‐based TCE over colorless polyimide via inkjet printing for capacitive touch sensing [137]. The TCE had 85% transmittance at 550 nm and a sheet resistance of 520 Ω/sq for a 70 nm layer thickness while demonstrating an invisible touch‐based security interface. Donaldson and Swisher developed an inkjet‐printed PEDOT:PSS TCE for multimodal neural sensing, with the TCE showing ∼77% transmittance at 510 nm while providing low‐impedance electrophysiological interfacing [134]. Similarly, Lee et al. printed a PEDOT: PSS‐based temperature sensor with >95% transmittance and sub‐millisecond temporal resolution, enabling good photothermal sensing and optical bio‐imaging [79]. Hao et al. fabricated an AgNW‐based flexible sensor via screen printing to detect liquid solvents [256]. Additionally, the cyclic stability of these electrodes was increased by applying a carbon coating on them. The resultant resistive sensor demonstrated varied current response on exposure to different solvents while still having transmittance of 77.6% at a visible wavelength of 550 nm. Finally, Noh et al. demonstrated a stretchable AgNW/PEDOT: PSS‐based TCE for biological signal processing [176]. The fabricated TCE had a sheet resistance of 25 Ω/sq at an optical transmittance of 85% and could acquire ECG, EMG, and EEG signals even from sensing areas below 1 cm^2^. These examples demonstrate the potential of printed TCEs for touch interfaces, neural monitoring, temperature sensing, and chemical/environmental sensing [116, 121, 129, 193, 198, 249, 252].

## 5 Concluding Remarks and Outlook

The development of TCEs is currently undergoing a dynamic transformation driven by the need for flexible, stretchable, and wearable electronic devices. To achieve the transformation, research in TCEs is shifting from identifying materials with high conductivity and transparency to engineering device‐compatible, manufacturable, and environmentally stable electrode systems. Materials such as metal meshes and nanowires, carbon materials, MXene, conducting polymers, and metal oxides have shown good transparency, low sheet resistance, and high flexibility, but have not yet replaced conventional ITO in practical electronics. Hence, further progress is required in the choice of TCE material, printing process, functional layers of the device, operating conditions, and device architecture. Thus, the selection of the optimum electrode is not a rigid universal selection but has to be tailored to the interfacial, chemical, mechanical, and processing requirements of the target device.

### 5.1 Device‐Specific Requirements

Several factors govern the performance of a device, and the most underestimated factor is the compatibility between TCE and the functional layers. In addition to sheet resistance and transmittance data, several other parameters like work function, surface energy, surface roughness, wettability, solvent resistance, thermal budget, chemical reactivity, and ion or metal migration are important considerations while selecting a TCE. For instance, in PSCs, the TCE must provide favorable energy‐level alignment with electron‐ or hole‐transport layers while remaining chemically compatible with the highly ionic perovskite absorber. Excessive surface roughness from metal meshes or nanowires can disrupt perovskite layers and increase the chance of shunting. Similarly, solvents used for fabricating transport or perovskite layers can dissolve or swell the underlying PEDOT:PSS layer. Similarly, Ag can react with halides under stress, while Cu needs an oxidation protection mechanism, and oxide‐based TCE may not have a suitable processing temperature for flexible polymer substrates while still being able to provide a planar surface.

In the case of OLEDs and PeLEDs, the requirements are more stringent due to having ultrathin charge transport and emissive layers. For instance, conductive protrusions or poorly embedded nanowire junctions can infiltrate other layers, creating short circuits and leakage paths leading to lower emissions. Similarly, PEDOT:PSS is good for work function tunability and surface planarization but has problems like acidity and hygroscopicity, reducing the stability of the other layers. In PeLEDs, the ionic perovskite may show halide‐metal interactions or field‐induced ion migration. Hence, electrodes with proper work function matching and chemically inert interfaces, along with effective encapsulation, are more important than ultralow sheet resistance.

In the case of photodetectors, electrode requirements are mainly guided by device architecture and operating wavelengths. Hence, high transparency is required over the spectral region of interest and not only at the wavelength of 550 nm. The TCE must allow the uniform deposition of photoactive materials while also minimizing dark current leakage. Photodetectors generally require high sensitivity and small variation in electrode morphology, or interfaces can influence noise, responsivity, and detectivity. Future studies should therefore evaluate wavelength‐dependent optical properties, contact resistance, interfacial trap formation, and temporal stability in addition to conventional sheet resistance/transmittance figures of merit.

A set of different constraints arised for transparent energy storage and electrochromic energy storage devices. Here, the TCE experiences electrochemical polarization, electrolyte contact, ion transport, and repeated volume changes in the active material. Hence, even though a TCE works well in solar cells or LEDs, it may fail in these systems. Metal corrosion, dissolution, electrochemical oxidation/reduction of conductive polymers, MXene oxidation, and loss of adhesion between active material and TCE can progressively increase interfacial resistance. Thus, a careful balance between optical transparency, electronic conductivity, electrochemical stability, electrolyte accessibility, and adhesion is required in future transparent energy‐storage electrodes.

For the case of wearable and stretchable electronics, selection is guided by mechanical and environmental compatibility. Hence, mechanical mismatch between rigid conductive layers and a soft elastomer can cause interfacial delamination. Thus, embedded metal meshes, serpentine structures, percolating nanowire networks, CNTs, hybrid electrodes, etc. are preferable for distributing strain.

### 5.2 Emerging Manufacturing and Process‐Controlled Directions

An important future direction is the transition from empirical printing optimization toward data‐driven and digitally controlled manufacturing. The printability of TCE inks depends on a variety of factors and thus makes conventional one‐factor‐at‐a‐time optimization inefficient. In this regard, machine learning ‐assisted ink formulation and process optimizations are slowly getting attention, where experimental datasets like droplet/filament formation, linewidth, roughness, conductivity, transparency, adhesion, and defect density could be used to identify processing windows and predict formulations for specific printing platforms. At present, the device TCEs are characterized only after full fabrication has happened using average sheet resistance and optical transmittance values. Problems start occurring when the electrodes are scaled as factors like localized discontinuity in the films, printing defects, pinholes, and variations in mesh width and thickness gradients are overlooked. A solution to this is the use of machine‐based systems with optical imaging, profilometry, electrical mapping, or spectroscopic monitoring for real‐time compensation of ink and other fabrication parameters to eliminate the defects. Another important aspect is the focus on large‐area roll‐to‐roll production for commercial manufacturing. Gravure, flexographic, screen, spray, slot/blade, and bar‐coating processes are naturally compatible with continuous manufacturing, while inkjet, EHD, and DIW techniques may provide complementary digital patterning where design flexibility or high resolution is required. Lastly, sustainability should become one of the key requirements of the fabrication process. Dependency on valuable materials like Au and In, intensive synthesis processes, and other additives needs to be cut down, and so future research should therefore consider recoverable and recyclable electrode architectures. Designing TCEs for disassembly and material recovery will become increasingly relevant if transparent and wearable electronics are produced at large scale.

### 5.3 From Laboratory Demonstrations to Industrial Manufacturing

A substantial gap between record‐level laboratory performance and industrially viable TCE production is still present. Laboratory studies are mainly limited to small‐scale or ‐area devices based on TCE, whereas manufacturing requires statistically reproducible performance across many devices and also sometimes on square‐meter‐scale substrates. Besides, variation in AgNW length distribution, CNT chirality and agglomeration, graphene defect density, MXene flake size distribution, and oxidation, PEDOT:PSS composition and nanoparticle concentration, and other parameters like solvent evaporation or rheology can produce significant variations in final electrode performance. Similarly, large‐area uniformity and defect tolerance should also become key performance metrics toward commercialization. Spatial mapping of sheet resistance, transmittance, haze, thickness, surface roughness, adhesion, work function, and defect density over representative device areas and their statistical distributions need to be reported rather than only best or average values. Also, apart from only calculating how surface planarity and embedded technologies decrease sheet resistance and performance, the effect of this on device yield is also an important criterion for commercial manufacturing. This is especially important in OLEDs, PeLEDs, and thin‐film photovoltaics where a small increase in defect density can lead to a reduced manufacturing yield. Another important aspect is that the encapsulation of the devices needs to be prioritized wherever needed, and development of TCEs with lower encapsulation requirements can be more economically attractive. While techniques like EHD and DIW can easily produce small and high‐aspect‐ratio features, their serial nature limits production, leading to lower throughput. Conversely, printing techniques like gravure and screen‐printing sacrifice on resolution and pattern flexibility while having higher throughput. Hence, techniques like multi‐nozzle printing have rapid curing, and hybrid combinations of analog and digital printing are important for increasing throughput while maintaining pattern accuracy.

Ultimately, the commercial success of the next generation of printed TCEs depends on balancing factors such as conductivity, transparency, and sustainability, rather than simply outperforming ITO. Future research should focus on integrating advanced manufacturing techniques and design principles to ensure reproducible and economically viable production, transitioning TCEs from laboratory demonstrations to critical components in flexible and wearable electronics.

## Author Contributions

**Vinayak Vitthal Satale**: writing – review and editing, writing – original draft, validation, software, resources, methodology, investigation, formal analysis, conceptualization. **Sourav Acharya**: writing – review and editing, writing – original draft, investigation, formal analysis, data curation, conceptualization. **Keum‐Jin Ko**: literature review, writing – original draft. **Sagnik Chowdhury**: literature review, writing – original draft and revision, software. **Danya Srinivasan**: literature review, writing – original draft. **Abrar Karim**: literature review, writing – original draft, Software. **Jae‐Wook Kang**: validation, supervision, software, resources, methodology, investigation, formal analysis, conceptualization.

## Conflicts of Interest

The authors declare no conflicts of interest.

## Data Availability

The data that supports the findings of this study are available in the supplementary material of this article.
